# The Long Non-coding RNAs: Paramount Regulators of the NLRP3 Inflammasome

**DOI:** 10.3389/fimmu.2020.569524

**Published:** 2020-09-25

**Authors:** Mridula P. Menon, Kuo-Feng Hua

**Affiliations:** ^1^Department of Biotechnology and Animal Science, National Ilan University, Yilan, Taiwan; ^2^Department of Pathology, Tri-Service General Hospital, National Defense Medical Center, Taipei, Taiwan; ^3^Department of Medical Research, China Medical University Hospital, China Medical University, Taichung, Taiwan

**Keywords:** lncRNAs, NLRP3 inflammasome, nuclear regulation, cytoplasmic regulation, chromatin remodeling, transcriptional regulation, scaffolds, decoys

## Abstract

The NOD LRR pyrin domain containing protein 3 (NLRP3) inflammasome is a cytosolic multi-proteins conglomerate with intrinsic ATPase activity. Their predominant presence in the immune cells emphasizes its significant role in immune response. The downstream effector proteins IL-1β and IL-18 are responsible for the biological functions of the NLRP3 inflammasome upon encountering the alarmins and microbial ligands. Although the NLRP3 inflammasome is essential for host defense during infections, uncontrolled activation and overproduction of IL-1β and IL-18 increase the risk of developing autoimmune and metabolic disorders. Emerging evidences suggest the action of lncRNAs in regulating the activity of NLRP3 inflammasome in various disease conditions. The long non-coding RNA (lncRNA) is an emerging field of study and evidence on their regulatory role in various diseases is grabbing attention. Recent studies emphasize the functions of lncRNAs in the fine control of the NLRP3 inflammasome at nuclear and cytoplasmic levels by interfering in chromatin architecture, gene transcription and translation. Recently, lncRNAs are also found to control the activity of various regulators of NLRP3 inflammasome. Understanding the precise role of lncRNA in controlling the activity of NLRP3 inflammasome helps us to design targeted therapies for multiple inflammatory diseases. The present review is a novel attempt to consolidate the substantial role of lncRNAs in the regulation of the NLRP3 inflammasome. A deeper insight on the NLRP3 inflammasome regulation by lncRNAs will help in developing targeted and beneficial therapeutics in the future.

## Introduction

### NLRP3 Inflammasome

The inflammasomes are a critical component of the innate immune system (1). They are multimeric intracellular protein complexes that comprises a sensor protein termed pattern recognition receptor (PRR) that oligomerizes and forms a platform to activate the pro-caspase-1 in response to the damage associated and pathogen associated molecular patterns (DAMPs and PAMPs) (2). Among the PRRs, the NOD LRR pyrin domain containing protein 3 (NLRP3) is crucial in host immune defenses against a broad range of infections. The NLRP3 cytosolic receptor is sensitive to a variety of cellular irritants, microbial components and endogenous alert signals such as ionic flux, reactive oxygen species (ROS), mitochondrial dysfunction and lysosomal damage (3). Their interaction with such stimuli results in the assembly and activation of the NLRP3 inflammasome that further activates caspase-1. The activated caspase-1 leads to the maturation and secretion of mature inflammatory mediator interleukin 1β (IL-1β), IL-18 and Gasdermin D. Activation of the NLRP3 inflammasome triggers pyroptosis, a caspase-1 dependent cell death, forces intracellular pathogens out of their replicative niche to the surroundings (4). The exposure of such pathogens triggers further cytokine release from other immune cells leading to the generation of DAMPs that additionally stimulate the immune cells to respond to the infection. The structural components, assembly and activation of the NLRP3 inflammasome have been reviewed in detail elsewhere (1–3).

The dysregulated NLRP3 inflammasome was associated with the pathogenesis of several autoimmune, autoinflammatory conditions, infections and tumorigenesis (5–7). The role of NLRP3 inflammasome in various pathogenesis has just begun to be explored. Their role in the development and progression of several diseases has been discussed in detail in Supplementary Table 1. A substantial amount of information on their involvement in diseases still remains to be comprehended. Undoubtedly, further clarified and precise understanding of the molecular mechanism behind the NLRP3 inflammasome regulation will guide the development of new effective therapeutics (8).

### Long Non-coding RNAs

The identification of non-protein coding RNAs as the key regulators of eukaryotic gene expression is a recent breakthrough. Approximately 80% of mammalian genome comprises non-protein coding regions that are transcribed into non-coding RNAs (9). Based on their length, the non-coding RNAs are classified into two categories: short non-coding RNAs (<200 nucleotides), such as microRNAs (miRNAs), and long non-coding RNAs (lncRNAs) (>200 nucleotides) (10). Based on their genomic locations, lncRNAs are grouped as long intergenic lncRNAs (lincRNAs), intronic sense lncRNAs and antisense lncRNAs. The biogenisis of messenger RNAs (mRNAs) and lncRNAs are the same for instance, lncRNAs also include transcripts derived from primary RNA polymerase II transcription (11). They are generally known as unconventional lncRNAs (12). Besides, different isoforms of lncRNA arise from the synonymous locus due to the fact that lncRNAs can also be free of alternative cleavage, splicing and polyadenylation (13–15).

The stabilization and maturation of such lncRNAs by RNase P generates a triple helix structured lncRNA with matured 3′ terminal carrying U-A-U sequence (16, 17). In order to prevent exonucleolytic deterioration, lncRNAs also forms circular closed structures through covalent bonding (18, 19).

LncRNAs are 200 nt long non-coding transcripts that performs various functions in diverse biological events (20–22). Some of the functions include regulation of protein translation, transcription and DNA synthesis (23, 24). Besides, they also regulate diverse pathological and physiological events (25–32).

Up until now, the significant role played by lncRNAs in regulating gene expression was unexplored. However, recent advancement in lncRNA research has reported that their involvement in diverse aspects of gene regulation in various cellular and biological processes is the root cause of various pathogenesis. LncRNA can influence the genetic information, for instance, transcription, mRNA availability, and stability, splicing, modulation of chromosomal structures and post-translational modifications (10). They carry out their regulatory functions through specific interactive domains for proteins, mRNAs, miRNAs, and DNA based on the secondary structures and sequences. They accomplish their function by acting as scaffolds, decoys, guides or signals (33).

Although lncRNAs coordinate several pathological events, their dysregulation causes severe pathologies. Various lncRNAs have been reported in the pathogenesis of inflammatory diseases such as arthritis, viral infections, intestinal disorders for instance celiac disease and breast cancer. They suppress or enhance the inflammatory responses in a gene dependent and time-dependent manner. Emerging shreds of evidence suggest that lncRNAs have critical involvement in the regulation of the immune system and the advancement of autoimmunity. Their expression in various immune cells such as macrophages, T lymphocytes, neutrophils, B lymphocytes, NK cells, and dendritic cells shows their importance in immune system maintenance. Reports suggest that lncRNAs are also involved in the differentiation and activation of these immune cells (33).

Keeping in view the emerging affirmation about the critical role played by lncRNAs in immune responses, in this review, we try to explore the involvement of lncRNA in regulating the activation and suppression of the NLRP3 inflammasome. We also provide the mode of action for how lncRNA regulates the NLRP3 inflammasome and drugs used to modify the function of lncRNAs in relation to the NLRP3 inflammasome. This is the first attempt being made so far to provide a consolidated review on the contribution of lncRNAs in managing the most important inflammatory machinery, the NLRP3 inflammasome.

#### Role of LncRNAs in the Development of Immune Cells

**Figure 1:**
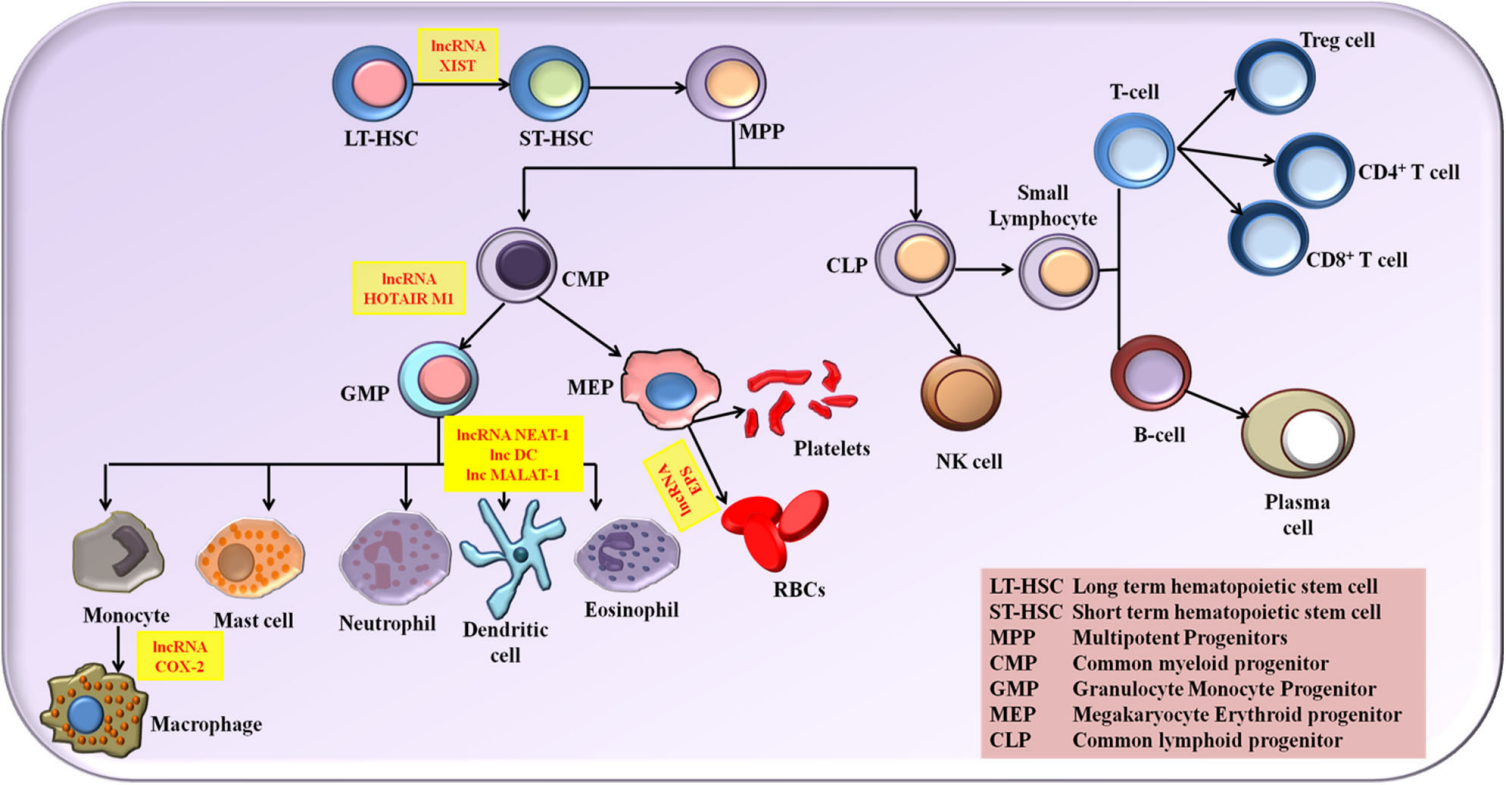
The NLRP3 inflammasome regulating lncRNAs in the activation and differentiation of myeloid and lymphoid cells.

The role of lncRNA in molding the immune response is grabbing the attention of researchers globally. Numerous studies put forth the fact that lncRNAs provide profound contribution in the immune cell lineage distribution and commitment (34). Myeloid cells comprising macrophage, monocytes and dendritic cells (DCs) and lymphoid cells comprising natural killer cells (NK cells), T cells and B cells are the two main classes of immune cells that are responsible for molding an immune response. The expression status of various lncRNA is associated with the growth, activation and differentiation of immune cells. Although lncRNAs linked to immune cell differentiation has been discovered, their complete functional profile is not yet elucidated. Based on the inflammatory conditions, lncRNAs has the potential to polarize the immune cells. Proper and in dept understanding of the influence of lncRNA in the differentiation and maturation of these immune cells provides us the benefit of exploiting the lncRNA regulatory potential in designing novel therapeutics and diagnostics. A detailed review on the roles of different lncRNAs in the immune cell differentiation has been explained elsewhere (34–38). In this review, we describe briefly the role of major NLRP3 inflammasome regulating lncRNAs in activation and differentiation of various myeloid and lymphoid cells (Figure 1).

## LncRNAs in NLRP3 Inflammasome Regulation

**Figure 2:**
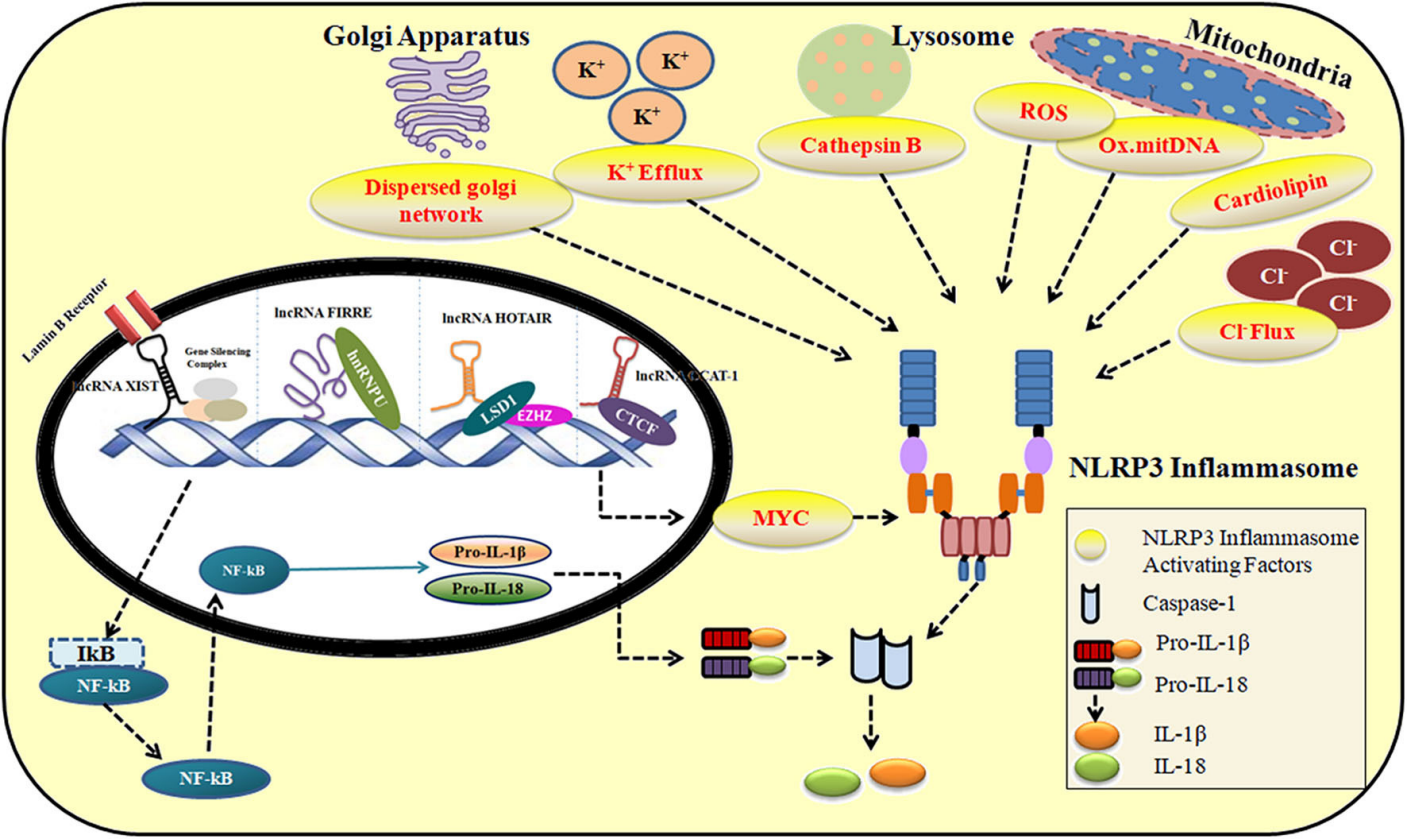
The role of lncRNAs in the activation and repression of NLRP3 inflammasome in response to multiple stimuli.

The role of lncRNAs in maintaining immune system homeostasis has largely been unrecognized. NLRP3 inflammasome machinery is critical to defending host cells from foreign infections. However, their unregulated activity results in life-threatening disease conditions as explained in Supplementary Table 1. In the following sections, we discuss the significant role of lncRNAs in regulating the activity of NLRP3 inflammasome under various conditions. Figure 2 describes the contribution of lncRNA in the activation and repression of NLRP3 inflammasome under multiple stimuli. Supplementary Table 2 provides a summary of lncRNAs regulating NLRP3 inflammasome activity.

### Positive Regulation of the NLRP3 Inflammasome by LncRNAs

#### LncRNA NEAT-1

The lncRNA NEAT-1 (nuclear enriched abundant transcript 1) is a nuclear lncRNA composed of transcripts with mainly two isoforms namely, NEAT1v2 of 23 kb and NEAT1v1 of 37 kb. These two isoforms of NEAT 1 are important requirements for the formation of nuclear paraspeckle and assist in their interaction with other paraspeckles proteins (39). NEAT 1 conciliate gene expression by transcription factor recruitment, sequestration, gene expression alteration and amending RNA splicing by interacting with RNA binding proteins (RBP) (40–45). The significant contribution of NEAT 1 in various neurodegenerative diseases, multiple viral infections and immune responses has been reported recently. Various studies also suggested major NEAT-1 dysregulation as the driving force for various disease progressions (46, 47). The link between lncRNA NEAT-1 and NLRP3 inflammasome behind multiple inflammatory responses started to unravel in the recent years. Zhang et al. reported that lncRNA NEAT-1 (nuclear enriched abundant transcript 1) positively regulated the NLRP3 inflammasome assembly and cytokine production. In their study, they identified that NEAT-1 promoted the assembly of the NLRP3 inflammasome and pro-caspase-1 processing in mouse macrophages by stabilizing the mature caspase-1 heterotetramers that assisted and promoted the processing of pro-IL-1β to mature IL-1β thereby causing pyroptosis. The authors observed that NEAT-1 interacted with the p20 domain of caspase-1 and promoted the assembly of NLRP3 inflammasome in IL-6 independent manner. NEAT-1 upregulation was also reported in cases where hypoxic conditions prevailed. Thus, NEAT-1 was also found to have an influence on NLRP3 inflammasome activation during hypoxia. More compelling evidence on the role of NEAT-1 on the NLRP3 inflammasome activation surfaced in 2019 where NEAT-1 deficiency in peritonitis and pneumonia mouse models dramatically reduced the inflammatory responses. Zhang et al. also demonstrated that knockdown of NEAT-1 led to a prominent reduction in the ASC oligomerization thereby impairing the NLRP3 inflammasome assembly. NEAT-1 also interfered in the Mitogen activated protein kinase (MAPK) signaling pathway and regulated the expression of chemokines and cytokines such as CXCL-10 and IL-6. In the same study, Zhang et al. reported that the stimulating factors for NEAT-1 coincided with the stimuli that activated NLRP3 inflammasome, for instance, ROS, hypoxia-inducible factors (HIFs), viral infections and tumor suppressor p53 (48). LncRNA NEAT-1 activates extracellular signal regulated kinase (ERK)/MAPK pathway in nucleus pulposus cells (NPCs) and induces the degradation of extracellular matrix (ECM) (49, 50). Xia et al. also reported the role of NEAT-1 in regulating the inflammatory cytokine expression including IL-1β, TNF-α and IL-6. Their study revealed that NEAT-1 significantly contributed in developing neuro pain through inflammatory cytokine expression in rat study model with chronic constriction injury (51). NEAT-1 also plays substantial role in tumor development and proliferation of cells (52)

In another study, Zhang et al. demonstrated that the expression of NEAT-1 was enhanced by 1.7-folds in dendritic cells (DCs) when stimulated with LPS for 24 h, and this effect was depending on TLR4-LPS interaction. Notably, NEAT-1 expression remained unchanged after stimulation of DCs with a series of ligands for TLR2, TLR3, TLR7, and TLR8 (53). It is well known that lncRNAs plays a crucial role in the activation and development of DCs (53). During chronic infections, the effective role of NLRP3 is significant for the development of CD11b+ DCs (54). Influenza A virus induced IL-1β and IL-18 secretion through the NLRP3 inflammasome in DCs (55). However, the involvement of NLRP3 inflammasome in tolerogenic DCs is a new area that started gaining more attention recently.

In the study, they determined that tolerogenic DCs were induced by the absence of NEAT-1. The ability of NEAT-1 to induce tolerogenic DC was confirmed by its knockdown in DCs. Such cells showed markedly reduced expression of co-stimulatory CD80, CD86, and MHC-II, reduced proliferation of T-cells, reduced the secretion of inflammatory cytokines IL-12, IL-17A, IL-1β, and IL-6 and increased number of Treg cells (53).

Tolerance in DC was promoted by the absence of NEAT-1 via regulating the NLRP3/IL-1β axis. NEAT-1 induced the expression of nlrp3 mRNA and protein. The expression levels of NEAT-1 was found to negatively correlate with the expression levels of miRNA, miR-3076-3p. NEAT-1 and NLRP3 was observed to be the direct targets of miR-3076-3p. The expression of nlrp3 mRNA and protein was observed to be reversed by the introduction of miR-3076-3p mimic (53).

It was observed that NEAT-1 sponged miR-3076-3p, inhibited their activity and acted as a ceRNA to modulate the NLRP3 expression. In murine models, NEAT-1 deficient DCs showed reduced inflammatory cell infiltration, decreased proliferation of T-cells and enhanced Treg cells (53).

#### LncRNA MALAT-1

LncRNA MALAT-1 (metastasis-associated lung adenocarcinoma transcript-1) is very well known for its functional and therapeutic competence. It is a highly conserved nuclear lncRNA that is confined to the nuclear speckles abundantly. One of the major functions of lncRNA MALAT-1 is to interact with the spliceosomal proteins (30, 56). With the half-life of 9–12 h and 3′ triple helix structure, MALAT-1 is the longest lncRNA. They are transcriptionally induced by several hormones and growth factors (57–60). LncRNA MALAT-1 also has a profound effect on NLRP3 inflammasome. Yu et al., reported the positive influence of lncRNA MALAT-1 on NLRP3 inflammasome expression in myocardial ischemia reperfusion injury (61). The mechanism by which lncRNA MALAT-1 exerts its influence on NLRP3 inflammasome expression is by acting as a competing endogenous RNA to sponge the inhibitory micro RNAs from inactivating the NLRP3 inflammasome for instance miR-133 and miR-203.

The association of lncRNA MALAT-1 with NLRP3 inflammasome was also successfully studied by Li et al. In their study, an enhanced expression of MALAT-1 was observed in macrophages obtained from diabetic mellitus and diabetic atherosclerotic rats (62). The involvement of MALAT-1 in the cell pyroptosis in the case of diabetic animals was reported by Han et al. (63). Their study demonstrated that overexpression of MALAT-1 improved the cell pyroptosis process, while knockdown of MALAT-1 significantly reduced the cell pyroptosis in macrophages (63). MALAT-1 assisted in enhanced expression of the NLRP3 inflammasome by sponging the miRNA, miR-23C. By sponging miR-23C, MALAT-1 prevented its inhibitory action on the target gene, ELAV1 (embryonic lethal abnormal vision-like 1). ELAV1 is an upstream gene associated with the NLRP3 inflammasome (63).

#### LncRNA COX-2

LncRNA COX-2 (cyclooxygenase-2) acquired its name due to its location with respect to the cyclooxygenase-2 gene. It is situated upstream 51 kb from the cyclooxygenase−2 gene. Recently there are various reports strongly suggesting the role of lncRNA COX-2 in diverse inflammatory activities and responses (64). Toll like receptors (TLRs) play a profound role in inducing lncRNA COX-2 to activate and inhibit various immune moldulatory genes (65).

In a study conducted by Xue et al. it was reported that lincRNA COX-2 also positively modulated the expression of NLRP3 and the adaptor protein ASC (66). Guttman et al. discovered that lincRNA COX-2 is situated at 51 kb upstream of Cox-2, the protein-coding gene (67). The TLR4 stimulation in CD11c+ bone marrow derived dendritic cells (BMDCs) induced 1,000-fold increase in the expression of lincRNA COX-2 (67). In bone marrow derived macrophages (BMDMs), TLR2 stimulation induced lincRNA COX-2 expression via MyD88 in NF-kB dependent manner (68).

Previously, it was reported that lincRNA COX-2 assists in the expression as well as repression of various immune genes in innate immune cells. However, Xue et al. managed to unravel the relation of lincRNA COX-2 with elevated expression of NLRP3 inflammasome and its associated proteins (66). LincRNA COX-2 assisted in enhancing the transcription of inflammatory genes by promoting the nuclear translocation of NF-κB p65. The translocation of these transcription factors into the nucleus led to the expression of NLRP3 inflammasome associated proteins (66). In macrophages and microglial cells, lincRNA COX-2 deficiency reduced the expression of NLRP3 protein and IL-1β secretion, whereas it enhanced the TRIF mediated autophagy (66). Autophagy removed damaged mitochondria that activated the NLRP3 inflammasome (66). TRIF cleavage is a crucial process that contributed to autophagy and is mediated by active caspase-1. Xue and co-workers showed that knockdown of lincRNA COX-2 reduced the expression of active caspase-1 and thus reduced the TRIF cleavage thereby promoting autophagy (66).

#### LncRNA ANRIL

Being at p21.3 in the CDKN2A/B human locus, the gene cluster containing lncRNA ANRIL (antisense non-coding RNA in the INK locus) also comprise three protein coding genes (69). RNA polymerase II transcription and tissue specific splicing results in various linear and circular isoforms of lncRNA ANRIL. The major functions of lncRNA ANRIL comprise interaction with histone modifiers especially polycomb repressive complex (PRC), sponging of microRNAs and assisting in RNA-RNA interaction. LncRNA ANRIL also has a profound effect on cellular adhesion, proliferation and apoptosis pathway (70–73). Recently, several evidences have emerged linking lncRNA ANRIL and NLRP3 inflammasome.

In a study, Hu et al. demonstrated that lncRNA ANRIL positively influenced the expression of NLRP3 inflammasome. ANRIL is a 3.8 kb long non-coding RNA that is highly expressed in mammalian tissues and organs such as the liver and lungs (47, 74). High levels of ANRIL expression was observed in the serum samples of uric acid nephropathy (UAN) patients as well as uric acid-treated tubular epithelial cells (HK-2). In these samples, the expression of ANRIL was elevated along with BRCA1 and BRCA2 containing complex subunit 3 (BRCC3). BRCC3 is reported to be a deubiquitinating enzyme that can reduce the level of NLRP3 protein ubiquitination thereby promoting the activation of NLRP3 inflammasome (75). Hence, ubiquitination regulation is considered to be the molecular switch in the activation of the NLRP3 inflammasome. However, in the same serum samples of UAN patients, the level of miRNA, miR-122-5p was simultaneously observed to be low. Hu and co-workers demonstrated that lncRNA ANRIL and BRCC3 shared the same miRNA response element with miR-122-5p (76). It was proved that in UAN pathogenesis, lncRNA ANRIL sponged miR-122-5p thereby inhibiting the negative regulation of BRCC3 by miR-122-5p. Thus, ANRIL positively regulated the activation of NLRP3 inflammasome in UAN conditions by promoting the expression of BRCC3 (76). The influence of ANRIL in renal injury was understood through *in vivo* studies. In the UAN rat model, the absence of ANRIL resulted in the suppression of renal injury by exhibiting an anti-apoptosis effect (76).

#### LncRNA KCNQ10T1

LncRNA KCNQ10T1 (KCNQ1 overlapping transcript 1 or KCNQ1 opposite strand anti-sense transcript 1) is one of the functionally well characterized lncRNA that is located in the KCNQ1 locus in humans along with 8–10 protein coding genes. lncRNA KCNQ10T1 assist in gene regulation process that are crucial for development and growth prior birth (77). However, only few studies have been reported on the influence of lncRNA KCNQ10T1 on NLRP3 inflammasome regulation. Jin et al. demonstrated that lncRNA KCNQ10T1 promoted cataractogenesis by enhancing the expression of active caspase-1 (78). Within the Kcnq1 region, KCNQ10T1 modified the expression of imprinted genes in a tissue-specific manner. In cataract formation, imprinted genes positively regulated the process of apoptosis (78). In cataract lens anterior capsule tissue samples and H_2_O_2_ treated SRA01/04 cells the expression of lncRNA KCNQ10T1 and active caspase-1 was up-regulated while the expression of miRNA, miR-214 was down-regulated. The expression of KCNQ10T1 correlated with the expression of caspase-1. Jin et al. further determined that KNCQ10T1 and caspase-1-3′UTR shared the same binding region with the miRNA, miR-214. In this study, it was established that the functional target of lncRNA KNCQ10T1 was miR-214 whereas the downstream target of miR-214 was caspase-1 (78). During cataract formation, the lncRNA KCNQ10T1 acted as a competing endogenous miRNA (ceRNA) sponge to miR-214 and interactively suppressed its inhibitory action on caspase-1. KCNQ10T1 subsequently promoted the activation of caspase-1 thereby causing pyroptosis (78). Although, evidences support the positive influence of lncRNA KCNQ10T1 on the activation of NLRP3 inflammasome, more studies needs to be performed to understand their contribution in various inflammatory diseases involving the NLRP3 inflammasome in detail.

#### LncRNA Gm4419

LncRNA GM4419 is known to promote diabetic nephropathy and cardiac diseases (79). In cerebral oxygen-glucose deprived/reoxygenation (OGD/R) condition, neuroinflammation is a prominent feature. Wen et al. reported that lncRNA Gm4419 played a significant role in the activation and progression of neuroinflammation during cerebral OGD/R (80). In microglial cells treated with OGD/R, the expression of Gm4419 was up-regulated along with inflammatory cytokines such as TNFα, IL-1β, and IL-6. NF-kB signaling cascade is crucial for inflammation (80). Gm4419 promoted NF-kB activation during neuroinflammation in microglial cells by assisting the nuclear translocation of the transcription factors p65 and p50. The lncRNA Gm4419 physically associated with IkBα and promoted its phosphorylation. This enhanced the availability of nuclear NF-kB for the transcriptional activation of inflammatory cytokines. Wen et al. also demonstrated that deficiency of Gm4419 contributed to NF-kB inhibition in OGD/R microglial cells (80). Similar effects of lncRNA Gm4419 on the activation of NLRP3 inflammasome during the progression of diabetic nephropathy (DN) were reported by Yi et al. (81). In their study, it was shown that the absence of Gm4419 reduced mesangial cell (MC) proliferation, pro-inflammatory cytokines, and renal fibrosis marker expression even in the presence of a high concentration of glucose. However, the overexpression of Gm4419 reversed these effects in MCs even in the presence of low glucose levels. Gm4419 interacted directly with p50, a subunit of NF-kB that interacted with NLRP3 inflammasome in MCs and promoted the progression of DN (81).

#### LncRNA MEG-3

LncRNA MEG 3 (maternally imprinted gene 3) is a pituitary cell expressed gene that is maternally imprinted. One of the mechanism by which lncRNA MEG 3 executes its function is by modifying the pathway of p53 (77). LncRNA MEG 3 enhances the protein expression of p53 by protecting it from spontaneous degradation mediated by the ubiquitin proteasome. lncRNA MEG 3 assist in the suppression of MDM2, an E3 ubiquitin liagase thereby blocking the ubiquitination of p53. This in turn encourages the interaction of p53 and target promoters thereby enhancing p53 dependent processes (82, 83). The link between lncRNA MEG 3 and NLRP3 inflammasome in various inflammatory conditions started emerging in recent years.

Atherosclerosis (AS) is an inflammatory disease caused by the pyroptosis of aortic endothelial cells. Zhang et al. reported that during AS in human aortic endothelial cell (HAECs) samples, the protein expression of NLRP3, ASC and inflammatory cytokines were relatively high (84). In the same samples, Zhang et al. also observed an up-regulated expression of lncRNA MEG-3 and down-regulated expression of the miRNA, miR-223. MEG-3 is a maternally imprinted lncRNA (84). The study indicated that the lncRNA MEG3 positively influenced NLRP3 inflammasome induced pyroptosis in HAECs during AS. MEG3 functioned as a ceRNA sponge and suppressed the inhibitory activity of miR-223 on NLRP3 inflammasome activation based on sequence complementarity (84). The segment spanning 498-518 in the MEG3 sequence was determined as the core region that interacted with miRNA, miR-223 and sponged its function. The study also showed that miR-223 overexpression evidently suppressed the pyroptosis of HAECs and the effect of miR-223 on pyroptosis was reversed by the overexpression of lncRNA MEG3 (84).

### Negative Regulation of NLRP3 Inflammasome by lncRNAs

#### LncRNA EPS

LncRNA EPS (erythroid prosurvival) is a long intergenic mammalian RNA that was identified in the year 2011. They are mainly associated with erythroid differentiation (85). Besides, they are also known to play a significant role in regulating the immune response gene transcription and positioning of nucleosome. Although they are abundantly expressed, their expression is significantly downregulated by LPS, the microbial ligand (86). Recently, various reports are surfacing in support of the role of lncRNA EPS as a host defense system during infections (87). LncRNA EPS is expressed in macrophage cells to regulate the expression of immune response genes (IRGs). Zhang et al. reported that lncRNA EPS was highly expressed in resting BMDMs while in TLR2 stimulated BMDMs, their expression was significantly down-regulated (84). The RNA levels of cytokines and chemokines such as Cxcl19, Cxcl0, Tnfsf10, Tnfsf8, IL-27, interferon-stimulated genes (ISGs) including Ifit2, Rsad2, Oasl1, and guanylate-binding protein (GBP) family members were altered with LPS stimulation in a time-dependent manner. Thus, lncRNA EPS executed strict control over IRG expression in a temporal manner (84).

The ectopic expression of lncRNA EPS on EPS deficient BMDMs curtailed the expression of IRGs which indicated the possibility of lncRNA EPS as a negative regulator of inflammation in macrophage cells (88). Microbial infections, endogenous danger signals, and environmental toxins lead to the activation of the NLRP3 inflammasome (89). LncRNA EPS expressing BMDMs exhibited impaired expression of IL-1β (88). Using the immunoblotting technique the authors showed that the level of ASC was significantly reduced in EPS expressing BMDMs compared to the control cells whereas the levels of pro- IL-1β, nlrp3, and pro-caspase-1 remained the same. LncRNA EPS regulated the expression of the pro-apoptotic gene, pycard that encoded the protein ASC (apoptosis-associated speck-like protein containing CARD) (88). ASC is a critical adaptor protein necessary for NLRP3 inflammasome assembly following its activation (85). Hence, lncRNA EPS negatively regulated the activation of NLRP3 inflammasome in unstimulated macrophage cells by suppressing the expression of the adaptor protein, ASC (88).

#### LncRNA Gm15441

LncRNA GM15441 is a transcript enriched in the liver that is suppressed during the progression of various metabolic diseases (90). They are mainly induced during the transition from feeding to fasting. LncRNA GM15441 imbricates *Txnip* gene. Due to this reason, the lncRNA GM15441 play a crucial role in energy consumption and metabolism (91, 92). The contribution of lncRNA GM15441 in regulating the NLRP3 inflammasome machinery in major metabolic disorders would be an interesting platform to explore. Recently, their involvement in NLRP3 inflammasome regulation has also been reported. Brocker et al. demonstrated that the induction of lncRNA Gm15441 attenuated the activation of hepatic NLRP3 inflammasome during metabolic stress through nuclear receptor peroxisome proliferator-activated receptor alpha (PPARA) (93). The process of fasting improves immunity by suppressing inflammation (93). Brocker et al. unraveled the molecular events that inhibited inflammation during restricted calorie intake. The PPARA plays a significant role during fasting by enhancing the consumption of lipids as an alternative energy source (93). The process of fasting activates PPARA which directly enhances the expression of lncRNA Gm15441. The lncRNA Gm15441 further restricts the expression of its antisense transcript, thioredoxin interacting protein (TXNIP). During oxidative and endoplasmic reticulum stress, TXNIP plays a critical role in inflammation by assisting in increasing the intracellular content of reactive oxygen species (ROS) (94–96). ROS is a well-known inducer and activator of the NLRP3 inflammasome. Thus, the suppression of TXNIP by lncRNA Gm15144 prevents the activation of NLRP3 inflammasome which in turn inhibits active caspase-1 generation and secretion of mature IL-1β, thereby preventing inflammation during fasting. The introduction of PPARA agonist to the Gm15441 knockout mouse model resulted in elevated expression of TXNIP, active caspase-1 as well as active IL-1β. This indicated that lncRNA Gm15441 is a significant negative regulator of the NLRP3 inflammasome by suppressing TXNIP (93).

#### LncRNA XIST

In mammals, lncRNA XIST (X inactivate-specific transcript) regulates the inactivation of X-chromosome. They also maintain the genome, monitors cancer cell differentiation and proliferation (97). LncRNA XIST functions as an oncogene and is de regulated in numerous non-sex linked tumors (98). Till date, their role in the progression and development of various types of cancers has been explored well (99–101). However, their association with NLRP3 inflammasome still needs to be elucidated. LncRNA XIST has been reported to play a critical role in the regulation of the NF-kB/NLRP3 inflammasome pathway in a negative manner during inflammation in bovine mammary epithelial cells (102). In the studies conducted by Ma et al. in bovine mastitic tissues and bovine mammary alveolar cell-T (MAC-T) cell models, up-regulated expression of lncRNA XIST was reported (102). Bovine mastitis or mammary gland inflammation is caused by the activated NF-κB which controls the cell proliferation, generation of inflammatory cytokines, for instance, TNF-α, IL-1β, IL-8, IL-6 and cell apoptosis (103, 104). The characteristic features of bovine mastitis include altered cell proliferation and migration (105), cell death, cytokine production (106), etc. It is a well-established fact that NF-kB is an upstream activator of the NLRP3 inflammasome. Ma et al. studies demonstrated that the inducing factors of the bovine mastitic conditions such as *E. coli* and *S. aureus* infections can, in fact, activate NLRP3 inflammasome and can successfully cause the related inflammatory responses (102).

In the bovine mastitis induced cell models, the expression levels of TNF-α, IL-1β, and IL-6 was also up-regulated along with lncRNA XIST (102). Gene silencing studies in inflammatory MAC-T cells resulted in reduced cell proliferation and elevated cell apoptosis in the absence of lncRNA XIST. The absence of XIST significantly enhanced the activation of NF-kB by promoting the phosphorylation of p65 and IkB as well as nuclear transport of the p65 subunit. Hence, lncRNA XIST suppressed NF-kB activation in inflammatory MAC-T cells (102). In addition, the silencing of XIST also increased the mRNA and protein levels of nlrp3, ASC, caspase-1, IL-18 and IL-1β in bacterial infection-induced inflammatory MAC-T cells. The administration of NF-kB signaling cascade inhibitor, BAY 11-7082 in MAC-T cells significantly reduced the transcriptional activation of lncRNA XIST. Hence, it can be concluded that the expression of lncRNA XIST during bovine mastitis is caused by the activation of NF-kB and lncRNA XIST negatively regulated the expression of NLRP3 inflammasome to prevent excessive inflammation in bovine mastitis (102).

### Mechanism of NLRP3 Regulation by lncRNA

LncRNAs are non-protein coding transcripts that exhibit specific regulatory functions, especially during inflammatory responses (21). LncRNAs control the gene expression of neighboring inflammatory protein-coding genes and thus contribute to the total inflammatory mRNA and protein concentration of the cell. LncRNAs have specific domains that can identify and interact with the target proteins and nucleic acid through complementary base pairing (21). This particular structural feature allows a single lncRNA molecule to connect with macromolecules of great diversity. The interaction of lncRNAs with the target proteins is mediated by ribonucleoprotein complexes (21). LncRNAs execute their regulation on inflammatory gene expression from various locations as they are localized within the cytoplasm, mitochondria, nucleus and nucleolus (107, 108). The localization of lncRNAs depends on the signature of the motifs for instance, Alu related sequences, protein signal peptides and nuclear-restricted lncRNA BMP/OP responsive gene (BORG) (109, 110). Besides localization, the secondary and tertiary structural configurations of lncRNAs are also significant for their mechanism of action (111). Each of these factors is described in detail in the following section.

#### Regulation at Different Cellular Locations

##### Regulation at the nucleus

LncRNAs are less conserved evolutionarily, not highly abundant and comprise fewer exons. All these features make lncRNAs different from mRNAs. Due to their high susceptibility to exosomal degradation on chromatin, inefficient polyadenylation and splicing, lncRNAs are mostly localized in the nucleus and they function as important nuclear organizers (9). The nuclear export of lncRNAs is prevented by the presence of *cis*-elements that are closely associated with nuclear proteins. A primate-specific short interspersed nuclear element (SINE) which is a C-rich sequence obtained from the Alu elements actively encourage the nuclear retention of lncRNAs by promoting the interaction with HNRNPK, a nuclear matrix protein. In some cases, the abnormal biogenesis pathways and the presence of specific *cis*-elements cause the nuclear accumulation of lncRNAs which has atypical forms (9).

**Figure 3:**
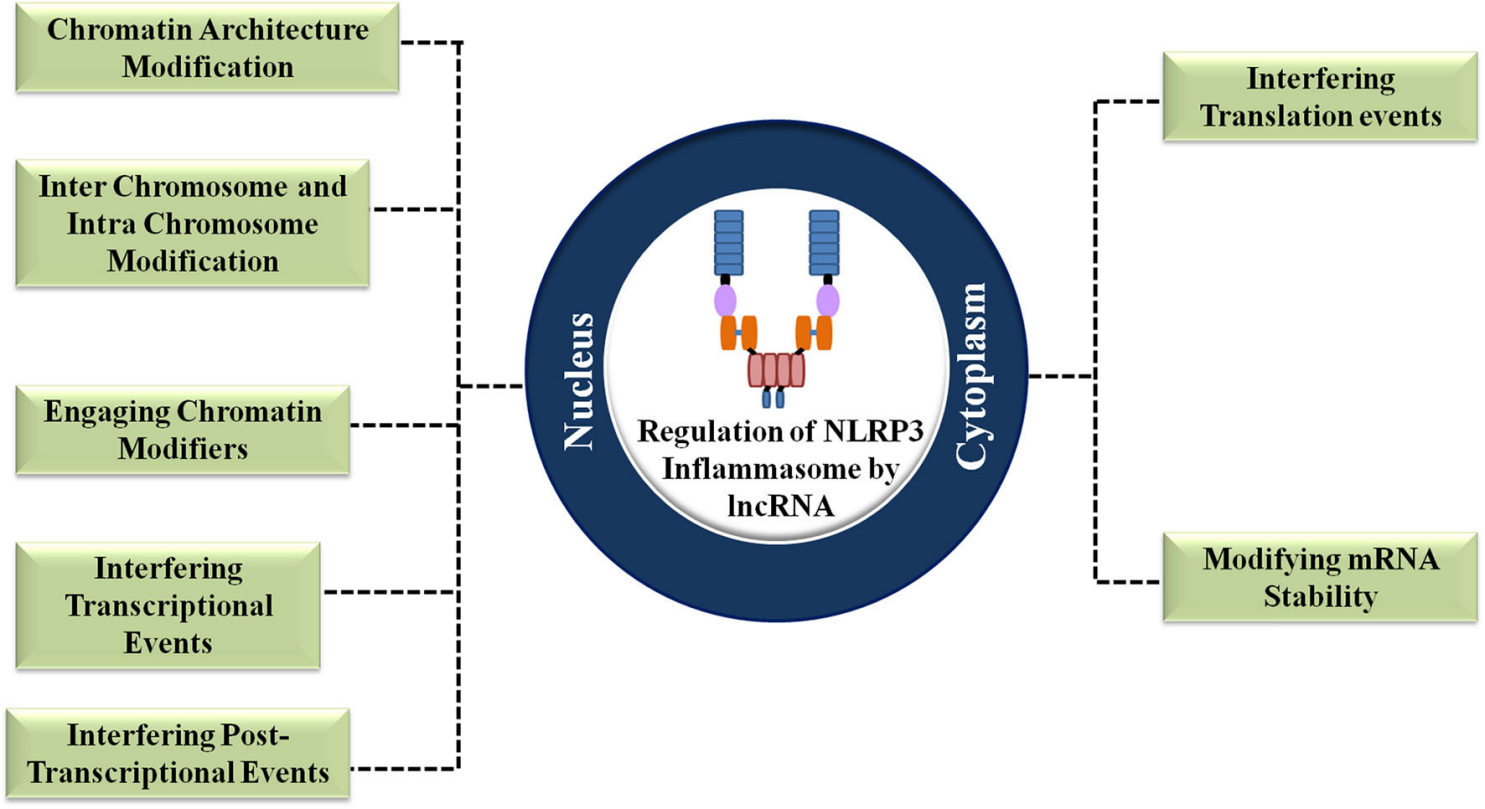
Regulation of the NLRP3 inflammasome by lncRNAs at different cellular locations.

**Figure 4:**
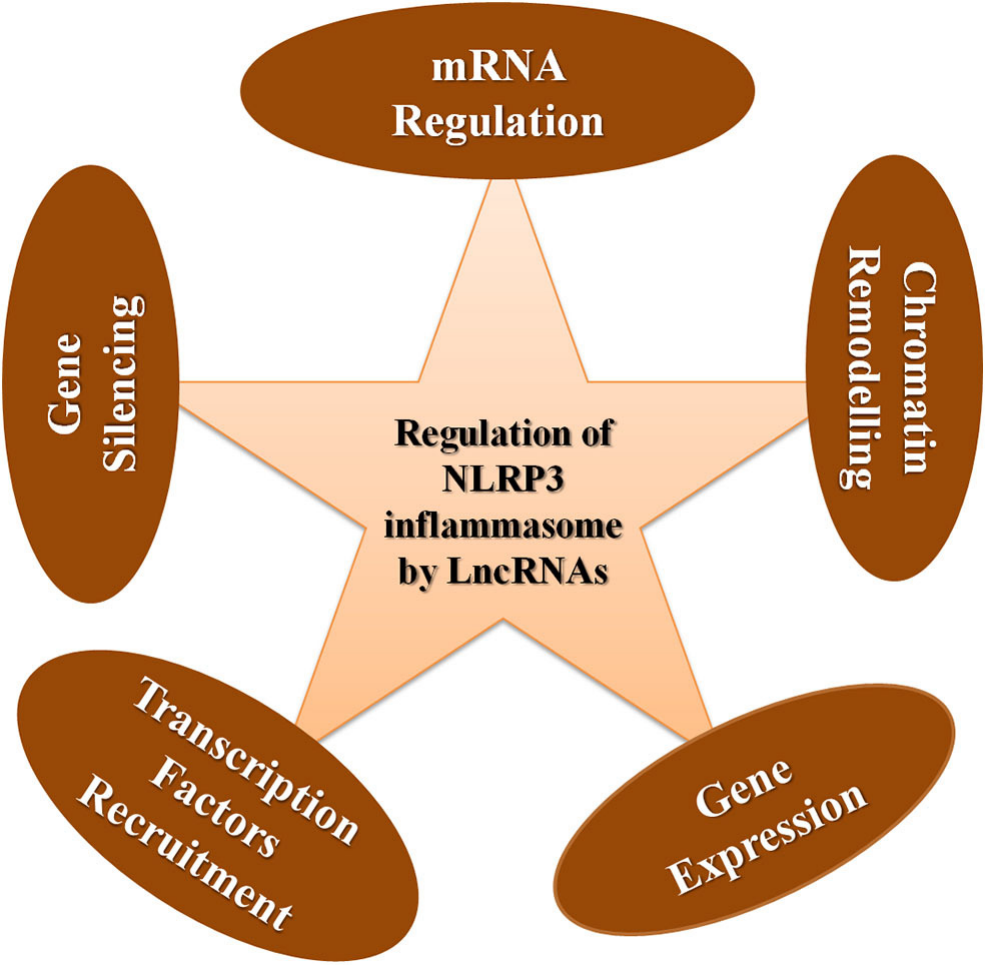
General ways involved in regulating the NLRP3 inflammasome by lncRNAs.

LncRNAs exert nuclear level regulation on NLRP3 inflammasome activation and the proceeding inflammatory responses in three levels (9). Firstly, they regulate the chromatin architecture and remodeling of inflammatory genes. Secondly, they regulate the transcription process of the inflammatory genes. Thirdly, they exert regulation post the transcription of inflammatory genes (9) (Figure 3). The general ways by which lncRNAs execute the regulation of NLRP3 inflammasome is shown in Figure 4.

##### Chromatin architecture modification

The highly organized interphase chromosomes provide an excellent platform for the lncRNAs to achieve NLRP3 inflammasome associated gene transcriptional regulation at different levels. The lncRNAs regulate the architecture of the chromosomes (112). The negative regulation of NLRP3 inflammasome by lncRNA XIST has been discussed in the previous section. LncRNA XIST regulates the expression of NLRP3 inflammasome at the chromatin level. Systemic Lupus Erythematosus (SLE) is an inflammatory condition that falls under category of autoimmune disorders. Yang et al. reported that the NLRP3 inflammasome activation and associated cytokine production are highly elevated in the macrophage cells of SLE patients (112). Strickland et al., reported the importance of proper X-chromosome inactivation in females and linked the X-chromosome abnormal inactivation with the high prevalence of SLE in females (113). Strickland and co-workers explained that there is an epigenetic event that silences most of the genes in one of the two X-chromosomes in females. The silenced genes may include DNA methylation, trimethylation, ubiquitylation, and histone deacetylation. However, during some instances, DNA methylation causes the abnormal activation of immune response-related genes in the X- chromosome which is supposed to be epigenetically inactivated. This induces SLE in females (9). LncRNA XIST plays a significant role in the X-chromosome inactivation in female mammals. The lncRNA XIST is transcribed from the X-chromosome which is supposed to be inactivated and their location is restricted across the entire X-chromosome. In order to achieve the stable silencing of the majority of X-chromosome, lncRNA XIST initiates series of signaling events that result in chromosome remodeling. The chromosomal condensation leads to the formation of heterochromatin Barr body of the inactivated X-chromosome which is localized to the nuclear lamina (9). LncRNA XIST is closely associated with the lamin-B-receptors in the nuclear lamina. Studies show that the disruption of lamin B receptor and lncRNA XIST interaction results in the impaired localization of inactivated X-chromosome followed by improper inactivation of the X-chromosome. This causes elevated expression of immune response genes due to DNA methylation which culminates in the activation of NLRP3 inflammasome and associated events in the case of SLE. In this way, lncRNA XIST modulates the chromosome architecture by recruiting the X-chromosome to the nuclear lamina which constrains the mobility of the chromosome. This facilitates the movement of lncRNA XIST and the assisting silencing protein complexes across the X-chromosome (9).

##### Inter chromosome and intra chromosomal interaction modification

The lncRNAs control the interchromosomal and intrachromosomal interactions to regulate the activation and expression of NLRP3 inflammasome associated genes. LncRNA functional intergenic repeating RNA elements (FIRRE) are transcribed from the X-chromosome and it interacts with the hnRNPU via its 156 repeating nucleotide motifs. Zhang et al. reported the upregulated expression of lncRNA FIRRE in the case of ischemic stroke in cerebral microglial cells through the activation of multiple inflammatory factors (114). The authors demonstrated that the upregulation of lncRNA FIRRE leads to the activation of NF-kB which activated the downstream signaling cascade that finally resulted in the activation of NLRP3 inflammasome (114). In the studies performed by Yao et al. it was shown that the lncRNA FIRRE accumulated near its transcription site (9). Using genome-wide mapping and RNA anti-sense purification in mouse embryonic stem cells, it was analyzed that lncRNA FIRRE established trans chromosomal contacts with additional five autosomal chromosomal loci and modulated the interchromosomal interactions to activate NLRP3 inflammasome by functioning as a scaffold (9).

The lncRNA colorectal cancer associated transcript-1 (CCAT-1) regulates the intra chromatin enhancer-promoter loops (115). In the upstream of the human MYC gene, colorectal cancer-specific super-enhancer is the transcription origin of lncRNA CCAT-1. The major function of lncRNA CCAT-1 is to promote the oncogenic activity and transcription of the MYC gene. Following the accumulation of lncRNA CCAT-1 at the transcription site, they interact with the CCCTC binding factor (CTCF) and bind together strands of DNA to form chromatin loops. This event localizes DNA to the nuclear lamina. The interaction of lncRNA CCAT-1 with CTCF results in the formation of a promoter-enhancer loop at the MYC locus thereby enhancing the transcription and oncogenic effect of the MYC gene. Zhao et al. demonstrated that the upregulation of the MYC gene is critical in inducing the NLRP3 inflammasome activation mediated proliferation of lymphoma cells (115). In this study, the authors showed that the activation of NLRP3 inflammasome in lymphoma is caused due to the upregulated expression of the oncogenic MYC gene and their expression is regulated at the nuclear level by lncRNA CCAT-1 (115).

##### Engaging chromatin modifiers

Several lncRNAs execute their regulation on NLRP3 inflammasome activation by remodeling the chromatin (9). These lncRNAs are localized in the nucleus and are chromatin-associated. They remodel the chromatin either in *trans* or in *cis* by promoting or preventing the chromatin modifier recruitment. The association between various lncRNAs such as HOTAIR, XIST and KCNQ10T1 and chromatin remodeling complexes are reported in numerous molecular studies (33, 116–118). Some of the histone modification events performed by lncRNAs in association with polycomb repressive complex 1 and 2 (PRC1 and PRC2) comprises histone 2A lysine 119 mono ubiquitinylaiton (H2AK119ub) (119), dimethylation and trimethylation of lysine 27 present in histone 3 (H3K27me3 and H3K27me2) (120, 121), demethylation of lysine 4 in histone 3 which is monomethylated and dimethylated (H3K4) (122) and histone methyltransferase (HMT) activity by G9a (123).

Several studies also reported that the absence of lncRNAs causes unsuccessful gene silencing (124–127). This emphasizes the importance of lncRNAs in navigating PRCs toward specific target site to perform their silencing. The target gene specificity of PRCs is determined by the type of lncRNA they associate with. For instance, the association of PRCs with lncRNA HOTAIR helps in targeting the locus HOXD (125) while, the interaction of PRC with lncRNA KCNQ10T1 targets the clusters in *kcnq1* (128). In another study, it was shown that interaction of lncRNA XIST with PRC target X-chromosome for histone modification (126). Although, PRCs can complex with various lncRNAs, further detailed molecular studies are required to understand in depth about their interacting domains specificity.

The ability of lncRNAs to modify histone and chromatin structure are utmost important in regulating gene expression associated with NLRP3 inflammasome during various inflammatory conditions. In SLE, extensive activation of NLRP3 inflammasome is reported. This is caused due to the accidental expression of the immune genes in the inactivated X-chromosome (9). LncRNA XIST is involved in the establishment of a repressive chromatin state across the X-chromosome that has to be inactivated by recruiting the chromatin modifiers. The transcribed lncRNA XIST interacts with hnRNPU and CIZI and remains closely associated with the X-chromosome that has to be inactivated. Further, this lncRNA directly recruits PRC2 components and assist their deposition at H3K27me3. LncRNA XIST mediates the stepwise recruitment of chromatin modifiers to the X- chromosome to inactivate them. The 4 Kb region present in the downstream of exon-1 of lncRNA XIST interacts with the non-canonical polycomb group RING finger 3/5 (PCGF 3/5)-PRC-1 complex (9). This allows the ubiquitylation of H2AK119u1 followed by the recruitment of other PRC1 complexes and PRC2.

The elevated expression of lncRNA HOX Transcript Antisense Intergenic RNA (HOTAIR) has been reported in the macrophages stimulated with LPS (129). Obaid et al. reported that the lncRNA HOTAIR interacts with the chromatin modifiers such as histone methylase (EZHZ) containing PRC2 complex and histone H3K4 demethylase (LSD1). LncRNA HOTAIR also interacts with PRCs to silence various inflammatory genes (125). They facilitate the gene suppression by recruiting LSD1 and PRC2 to the target genes (129). The lncRNAs have tremendous potential to modify the histones to regulate the expression of inflammatory genes. Tong et al. reported the role of lncRNA COX-2 in regulating gene expression through histone modification. The authors showed that lincRNA COX-2 expression in TNF-α stimulated intestinal epithelial cells, suppressed the expression of *Il12b* by recruiting the nucleasome remodeling and deacetylase repressor complex, Mi-2-NuRD at *Il12b* gene promoter region. Tong et al. further noted that lincRNA COX-2 assisted in histone modification epigenetically by reducing the methylation of H3K27 and enhancing the acetylation of H3K27 and H3K9. Their study also demonstrated that the histone modification events at *Il12b* gene promoter were attenuated by the knockdown of lincRNA COX-2 (117).

Once lincRNA COX-2 is induced in macrophages upon LPS stimulation, they are assembled on switch/sucrose non-fermentable (SW1/SNF) complex. In this way lincRNA COX-2 assist in chromatin remodeling and modulate the primary response gene transcription process when the cells encounter a microbial attack or when cells are stimulated with LPS (118). In a study, Pandey et al. reported the interaction of G9a HMT with PRC2 and lncRNA KCNQ10T1 (128). Similarly, Terranova et al. observed that PRC1/2 complex members and lncRNA KCNQ10T1 shared close proximity (130). Imprinting studies performed on mouse extra embryonic placental tissues revealed PRC2-G9a HMT induced repression on histones, K3K9me3 and H3K27me in *kcnq1* and *Igf2r* imprinted gene clusters (128, 131).

In many instances, the lncRNAs can restrict the recruitment of chromatin modifiers and inhibit the interaction between the specific DNA loci with the chromatin modifier. In such cases, the lncRNAs function as decoys (9). Several lncRNAs whose function has been explored as decoys have been reported, for instance, lncRNA Mhrt and lncRNA PRESS1 (9). However, the role of lncRNAs as decoys of chromatin modifiers in NLRP3 inflammasome regulation has to be explored further to conquer thorough understanding.

##### Interfering transcriptional events

In addition to the regulation on chromatin architecture, lncRNAs also regulate the expression of NLRP3 inflammasome and associated proteins at the transcription level (132). They form R-loop structures that assist in the recruitment of the respective transcription factors. R-loops are formed by the hybridization of RNA with the DNA duplex thereby forming a triple-stranded structure of nucleic acid (132). The R-loop structure secures the lncRNA in *cis* to the target gene thereby promoting the binding of transcription factors with the target gene promoter region. For instance, the transcription of lncRNA KHPS1 is in an anti-sense direction to that of the sphingosine kinase-1 (SPHK1) gene. SPHK1 is a proto-oncogene that catalyzes the phosphorylation of sphingosine to sphingosine-1-phosphate (132). Frevert et al. showed that the expression of SPHK1 enhanced the production of inflammatory cytokines CCL5 and CCL2 (132). However, studies reported that the expression of SPHK1 negatively regulated the expression of NLRP3 inflammasome and associated secretion of mature IL-1β by decreasing the availability of the DAMP, sphingosine. LncRNA KHPS1 plays a significant role in the activation of SPHK1 (132). The R-loop structure formed between the lncRNA KHPS1 and SPHK1 tethers KHPS1 in the upstream of the SPHK1 transcription start site (TSS). This recruits the histone acetyltransferases p300/CBP through lncRNA KHPS1 to the promoter region of SPHK1 thereby enhancing the chromatin accessibility for the binding of E2F1 (9). These events lead to the expression of SPHK1. Thus, lncRNA KHPS1 assists in the negative regulation of NLRP3 inflammasome by restricting the availability of sphingosine at the transcription level.

Another classical example of lncRNAs regulating the NLRP3 inflammasome expression in the transcription level is the expression of human gene vimentin (9). Vimentin is a type III intermediate filament protein that plays a critical role in intracellular architecture stabilization. Vimentin is also involved in enhanced cell invasion by improving cell migration (9). Santos et al. demonstrated the regulatory role of vimentin in the expression and activation of NLRP3 inflammasome (133). Vimentin assisted the build-up of NLRP3 inflammasome machinery as a scaffold and facilitated the maturation of IL-1β in pulmonary fibrosis and lung inflammation (133). Vim gene accommodates an anti-sense lncRNA VIM-AS1. Near the TSS of vimentin, an R-loop is formed by the lncRNA VIM-AS1 that promoted the transcription of vimentin by favoring the interaction between promoter and the transcription factor, NF-kB induced by local chromatin decondensation (9). Thus, lncRNA VIM-AS1 positively regulated vimentin expression which further promoted the NLRP3 inflammasome activation.

At the targeted gene loci, the lncRNAs interfere with the pol II transcription complexes to execute their control on the gene transcription (134). The lncRNAs impede with the Pol II complex and regulate the process of transcription elongation. The 7SK RNA is a pol III transcribed lncRNA that obstructs the pol II-mediated elongation by binding to p-TEFb, a positive elongation factor (134). The interaction between lncRNA 7SK-RNA and p-TEFb reduces its kinase activity that is critical for the positive elongation process during transcription. p-TEFb is a positive regulator of NF-kB (134). Upon NF-kB activation, p-TEFb causes the phosphorylation of pol II that assists in the dissociation of DSIF from the TATA rich promoter region of the NF-kB target gene. This switches the fate of NF-kB from DSIF to p-TEFb. The activated NF-kB positively regulates the expression of numerous immune response genes including NLRP3, pro-IL-1β, and pro-caspase-1(134). Hence, the lncRNA 7SK-RNA controls the availability and activation of p-TEFb which is necessary for the activation and expression of NF-kB dependent immune genes during inflammation.

The lncRNAs also regulate the pol I assisted transcription process (135). Pol I transcribes the ribosomal RNA (rRNA) from rDNA. rRNA is involved in the cellular protein synthesis that is important in various stages of cell growth processes. Several disorders with an unusual translational pattern are caused by uncontrolled pol I transcription (135). Several studies show that the functions of the ribosome when suppressed have the potential to activate the NLRP3 inflammasome (136). The inhibition of ribosomal translation triggers a stimulus for the activators of NLRP3 inflammasome. In addition, the suppressed translational process acts as a converging center for the signals released from pathogens, toxins and metabolic irregularities that trigger the activation of NLRP3 inflammasome and releases the mature IL-1β. Hence, regulated transcription of ribosomal components by pol I is highly necessary to ensure controlled inflammatory responses via NLRP3 inflammasome (136). The nucleolus localized lncRNA SLERT (snoRNA-ended lncRNA enhances preribosomal RNA transcription) regulates the function of pol I transcription machinery. Under demanding conditions, the lncRNA SLERT interacts with the RNA helicases DDX21, causing a conformational change in DDX21 that releases pol I from its inhibitory action. This enhances the pol I based transcription of rDNA. Thus, lncRNA SLERT indirectly regulates the activation of the NLRP3 inflammasome by repressing pol I from DDX21inhibitory action (136).

##### Interfering post-transcriptional events

LncRNAs regulate the expression of NLRP3 inflammasome associated genes at the post-transcriptional level as well. LncRNAs form membrane-less, dynamic nuclear bodies by forming complexes with proteins thereby controlling their coherence and functions (9). In the nuclear bodies, lncRNAs function as architectural and non-architectural RNAs to execute their function (9). Architectural lncRNAs act as the core of nuclear bodies by forming scaffolds. Nuclear bodies contain numerous paraspeckles that are formed and maintained predominantly by lncRNA NEAT 1. According to electron and structured illumination microscopic analysis, paraspeckles are spheroidally organized structures that are present in the interchromatin region which require the activity of both NEAT 1 and NEAT 2 (9). The inner core of the paraspeckles is made up of the middle region of NEAT 2 while the outer shell comprises the 5' end of NEAT 1 and both termini of NEAT 2. The stress-induced expression of lncRNA NEAT-1 dictates the morphology and count of the paraspeckles formed (9).

LncRNA NEAT-1 can regulate the activity of NLRP3 inflammasome in both nuclear and cytoplasmic levels. The cytoplasmic regulation of NLRP3 inflammasome by lncRNA NEAT-1 will be explained in the subsequent section. In the paraspeckles, lncRNA NEAT-1 sequesters >40 proteins, many of which have significant immune functions. For instance, the protein splicing factor proline/glutamine-rich (SFPQ) is sequestered in the paraspeckles. Hirose et al. showed that stress-induced expression of lncRNA NEAT-1 leads to specific protein sequestration in the paraspeckles to facilitate the target gene transcription (137). The inhibitory interaction of SFPQ with the target gene promoter is prevented by its sequestration in the paraspeckles. SFPQ inhibits the transcription and expression of IL-8 by binding to its promoter region and blocking its transcription (137). However, stress-induced expression of lncRNA NEAT-1 enhances the expression of IL-8 by relocating and binding SFPQ in the paraspeckles thereby facilitating the access of IL-8 promoter region to the transcription machinery (41, 138). IL-8 is one of the inflammatory cytokines secreted by the active caspase-1targets, IL-1β and IL-18 (41, 138). Thus, lncRNA NEAT-1 indirectly promotes the inflammatory environment prevailing in the host which is generated by the active NLRP3 inflammasome target cytokines, IL-1β and IL-18.

In the nuclear bodies, lncRNAs also contribute to the regulation of NLRP3 inflammasome activity in a non-architectural manner (9). As mentioned in section LncRNA MALAT-1, lncRNA MALAT-1 positively regulated the expression of NLRP3 inflammasome. In the nuclear bodies, the region with an abundance of pre-mRNA is called nuclear speckles. The mature lncRNA MALAT-1 is a stable 7,500 nucleotide triple helical structure containing a 61 nucleotide mascRNA (MALAT-1 associated small cytoplasmic RNA) which is formed after an RNase P mediated processing of its 3′ end (9). The periphery of the nuclear speckles contains small nuclear RNAs (snRNAs) and MALAT-1 whereas its central region comprises SON and SC35 proteins. In the nuclear speckles, the interaction of MALAT-1 with RNA exporting and RNA splicing SR proteins help them to execute their regulatory function by impacting the process of splicing. After being recruited to the nuclear speckles, MALAT-1 interacts with the pre-mRNAs that are alternatively spliced via a protein mediator. MALAT-1 promotes protein-protein, protein-DNA, and protein-RNA interaction by acting as a scaffold within the nuclear speckles (9).

##### Regulation at the cytoplasm

LncRNAs are also translocated into the cytoplasm to directly or indirectly regulate the activity of NLRP3 inflammasome. In the cytoplasm, they supervise the stability of mRNA, inflect the process of translation and intervene with the events of post-translational modifications (PTMs) (Figure 3).

##### Modifying the mRNA stability

There are several ways by which lncRNAs regulate the stability of NLRP3 inflammasome associated mRNAs. They function as competitive endogenous RNAs (ceRNAs) and sponge the miRNA by competing for their binding sites (9). By sponging miRNAs, the ceRNAs derepresses the targets of miRNA (9). LncRNA Cyrano sponges the activity of miR-7 and releases the nlrp3 mRNA from its inhibitory effect (139). miR-7 inhibits the translation of nlrp3 protein by occupying the UTR domain of the nlrp3 mRNA. The 5' UTR and 3′ UTR regions play a significant role in the translation of mRNA (139). The termination of transcription and post-transcriptional gene expression heavily rely on the 3′ UTR region of an mRNA whereas 5' UTR is the leader sequence in which the first codon for translation is present (139). Hence, blocking the UTR region in an mRNA intervenes with the effective translation process. LncRNA Cyrano acts as a ceRNA and facilitates the tailing and trimming of the 3′ end of miR-7 which leads to miR-7 degradation (139). Thus, lncRNA Cyrano stabilizes the mRNA of nlrp3 and promotes the NLRP3 inflammasome mediated inflammatory action.

LncRNA NEAT-1 assists in the activation of NLRP3 inflammasome in mouse macrophages. They aid in NLR3 inflammasome assembly, processing and stabilization of caspase-1thereby promoting IL-1β triggered pyroptosis (48). Upon receiving NLRP3 inflammasome stimulating signals such as LPS and Nigercin, the nuclear paraspeckles containing lncRNA NEAT-1 is weakened and disrupted to export NEAT-1 to the cytoplasm. There they support the assembly of the NLRP3 inflammasome by colocalizing with the adaptor molecule ASC. They interact with caspase-1 p20 domain and stabilizes the (p20:p10)2 hetero-tetramer (48). Hence, lncRNA NEAT-1 exert their regulatory function on NLRP3 inflammasome dependent inflammatory responses in both nuclear and cytoplasmic level.

The lncRNA, non-coding RNA activated by DNA damage (NORAD) regulates mRNA stability by acting as molecular decoys (9). mRNA degradation occurs by the interaction of PUMILIO 1 and PUMILIO 2 (PUM1/2) protein with the 3′ UTR region of the target mRNA. This results in decapping and deadenylation of the target mRNA which ultimately hinders the translation process (9). During replication stress and DNA damage, lncRNA NORAD enhances the genomic stability by binding and sequestering PUM1/2 in the cytoplasm. This restricts their availability to degrade the target mRNAs (140). The targets of PUM1/2 are mainly mRNAs that are crucial for DNA replication, mitosis, and DNA damage repair. Numerous studies have reported the direct link between DNA damage and activation of NLRP3 inflammasome. The stimulation of NLRP3 inflammasome activity by DNA damage results in p53 activation and associated innate cell apoptosis (141). The study conducted by Chaudary and Lal sheds light on the effect of p53 activation in lncRNA NORAD (142). The authors reported the activation of several lncRNAs by p53 during DNA damage however; lncRNA NORAD is not a direct target of activated p53 (142). NLRP3 inflammasome promotes p53 mediated cell death by the annihilation of DNA repair (143). However, a direct relation between lncRNA NORAD and NLRP3 inflammasome activation during cell stress-induced DNA damage has not yet been explored.

##### Interfering translational events

LncRNAs do not exhibit translational capacity however; they are involved in regulating the process of translation (144). LncRNAs play crucial role in promoting mRNA translation of inflammatory genes. They can regulate immune gene expression at translational as well as post-translational levels. Direct interaction of lncRNAs with multiple proteins enables their regulatory function. They modulate mRNA translation by binding to the translation ignition complex (145). LncRNAs also significantly modulate post translational events including protein acetylation, phosphorylation and ubiquitination.

The protein production in a cell is always affected directly by the mRNA abundance. In general, the decay rate and transcription rate of mRNA indicates the mRNA abundance in the cell (145). Recently, several studies on lncRNAs have reported their potential role in executing mRNA decay (146). Besides mRNA decay, lncRNAs also regulate methylation and stability of mRNAs. The polypeptide coding and relocalization of proteins also comes under the influence of lncRNAs (146). The potential of lncRNAs to modulate translation and post translation events has been reviewed in detail elsewhere (145, 147, 148). To avoid redundancy, in this review we focus on lncRNAs ability to regulate translation of genes related to NLRP3 inflammasome.

The lncRNA MEG3 present in human chromosome 14q32.3 at locus Dlk1-Gtl2 plays important role in mRNA decay. The LC-MS and RNA pull down studies performed by Zhou et al. showed that lncRNA MEG3 regulates complete metabolism of mRNA by interacting with polypyrimidine tract binding protein 1 (PTBP1). The lncRNA bound PTBP1 binds to the mRNA small heterodimer partner (SHP) at the CDS region. This resulted in the decay of SHP mRNA (149, 150). In another study, Yoon et al. showed that LincRNA-p21 binds to an RNA binding protein, HuR and inhibits its effective functioning in mRNA stabilization (144). The association of lincRNA-p21 and HuR triggers the entry of miRNA let-7 and the protein argonaute-2 (Ago-2) which synergistically acts on the lincRNA-p21-HuR complex and dissociates lincRNA p-21. The released lincRNA-p21 further binds and suppresses the translation of CTNNB1 and JUNB mRNAs via repressor Rck (144). JUN B is a transcription factor that plays a critical role in macrophage immune activation. In LPS activated macrophages, JUN B is required for the complete expression of the interleukin-1 family of cytokines (151). Hence, lincRNA-p21 alleviates IL-1β mediated inflammatory responses at the translational level and indirectly controls the NLRP3 inflammasome induced responses in the host.

Similar to their role in preventing translation, lncRNAs also play a vital role in promoting translation (152). LncRNA antisense to uchl 1 (AS-Uchl1) promotes the translation of Uchl1 (ubiquitin carboxyterminal hydrolase L1) mRNA by active polysome generation in the mRNA. Within the 5' terminus of Uchl1 mRNA, the presence of a 73 nucleotide region complementary to the SINE B2 region assists in enhancing the lncRNA AS-Uchl1 mediated translation process (152). Uchl1 protein is involved in neurodegeneration in mice model. NLRP3 inflammasome activation is a significant factor in the onset and progression of the neurodegenerative conditions (152). Although a direct relationship between lncRNA AS-Uchl1 and NLRP3 inflammasome has not been discovered so far, with the available resources until now, it can be said that lncRNA AS-Uchl1 indirectly assist NLRP3 inflammasome mediated development of the neurodegenerative condition in the translation level (152).

There are several lncRNAs regulating the events in post-translation modification (PTM) to execute their function in various cellular processes such as DC differentiation and breast cancer metastasis reduction. However, only limited studies has been reported so far explaining lncRNAs regulatory action on NLRP3 inflamamsome via translation and PTMs. In conclusion, it can be stated that the versatile roles played by lncRNAs in regulating NLRP3 inflammasome activity mainly rely on their structure, interacting partners and subcellular localization.

## NLRP3 Inflammasome Regulators Taking Advantage of lncRNAs

NLRP3 inflammasome is a multicomponent inflammatory machinery that is mainly responsible for storming inflammatory cytokines. They are the most explored inflammasome mainly due to their ability to be activated by various stimuli (153). Gross et al. reported that recative oxygen species (ROS) can be a potent activator of NLRP3 inflammasome. Thioredoxin interacting protein (TXNIP) detects the difference in the concentration of ROS. Later, TXNIP dissociates from thioredoxin (TRX) to bind and activate NLRP3 inflammasome (154, 155).

Another potential stimulus regulating the NLRP3 inflamamsome activation is adenosine tri phosphate (ATP). In one of the studies by Bartlett et al. it was reported that the receptor P2X7 plays significant role in the activation of NLRP3 inflammasome in coordination with ATP (156). Yin et al. observed that P2X7 receptor detects increased extracellular ATP levels and produces ROS. This in turn activates NF-kB (157). Several acute myocardial infarction (AMI) studies conducted by Ward et al. confirmed the potential of P2X7 receptor in NLRP3 inflammasome activation (158, 159). In 2007 Petrilli et al. reported that P2X7 receptor activation resulted in intracellular K^+^ outflow. In this study, Petrilli et al. showed that reduced intracellualar K^+^ can effectively activate NLRP3 inflammasome (160).

Lysosomal rupture can also act as a potential NLRP3 inflammasome activation regulator. Upon encounter with DAMPs and PAMPs, phagocytosis occurs and lysosomes loses their integrity. NLRP3 inflammasome is activated upon their interaction with cytoplasmic hydrolysed lysosomal components (161, 162). Autophagy and mitochondrial autophagy are also important regulators of NLRP3 inflammasome. Yuan et al. reported that the dysfunction of Lysosome induced by CD38 knockout and inhibition of V-ATPase resulted in autophagy disorder which activated NLRP3 inflammasome (163).

Liu et al. discovered that sirtuin 3 (Sirt3) knockout inhibited autophagy and hence, resulted in activated NLRP3 inflammasome (164). THP cells overexpressing Sirt3 showed palmetic acid induced reduction in ROS production. In such cells, activation of NLRP3 inflammasome was reduced mainly due to the autophagy recovery. However, Lai et al. showed that mitophagy can inhibit NLRP3 inflammasome activation and hence acts as a negative regulator (165). Various studies demonstrated that PIK3CA-AKT–mTOR-RPS6KB1 pathway and AMPK/ULK1 pathway induced mitophagy results in inhibition of NLRP3 inflammasome activation (166, 167). Besides, various post translational modifications such as phosphorylation of ASC protein by spleen tyrosine kinase (syk) (168), dephosphorylation of NLRP3 PYD by phosphatase 2A (PP2A) (169), deubiquitination by BRCA1 and BRCA2 containing complex subunit 3 (BRCC3) (75) and linear ubiquitination also acts as potential NLRP3 inflammasome regulators (170).

Although various NLRP3 inflammasome regulators have been studied and reported, specific role of lncRNAs in assisting these regulators to activate and suppress NLRP3 inflammasome is still not completely explored. More studies are required to understand how lncRNAs are involved and utilized by these regulators to perform their functions. Supplementary Table 3 shows details on available lncRNAs influencing the regulators of NLRP3 inflammasome.

## Potential Drugs Targeting LncRNAs Involved in the NLRP3 Inflammasome Regulation

LncRNAs are non-coding RNAs that can regulate gene expression in diverse ways to execute cellular function. They carry out their role in gene expression modulation by interacting with various proteins, RNA and DNA counterparts. The abnormal presence of lncRNAs in various diseases and the emerging evidences on their functional implications in multiple biological pathways emphasizes the need to target them in modern medicine. Till date, lncRNAs are linked with multiple diseases and it is possible to target them as therapeutic strategy for instance, lncRNA H1F1A and MIAT during cardiovascular diseases (171), lncRNA HYMA1 and PINK1-AS during diabetes (172), lncRNA FMR4, HELLPAR, KCNQ10T1 and DBET during numerous genetic disorders (173), lncRNA TUNA, naPINK1, DISC2 and BACE1-AS for neurological disorders (174), lncRNA HOTAIR, H19, GAS5, MALAT-1 and XIST during various types of cancers and inflammatory conditions (175). During therapies, it is possible to target the complex structural residue formed between lncRNAs and the multi-component molecular structures (176).

Generally, there are several ways to target lncRNAs such as siRNA treatment (177), use of natural antisense transcript (NATs) (178), anti-sense oligonucleaotide (ASO) treatment (179), targeting lncRNAs with locked nucleic acid nucleotides (180), use of catalytic degraders for instance, ribozymes and deoxyribozymes (181), peptide aptamers (182), use of small molecule inhibitors that has the potential to mask lncRNA binding site (183), use of RNA destabilizing elements (RDEs) (184) and synthetic mimics (185). The role of each of these inhibitors in blocking the activity of lncRNAs are reviewed in detail elsewhere (176). In this review we focus mainly on the drugs that has the potential to control lncRNA function related to NLRP3 inflammasome involved inflammatory conditions.

Long non-coding RNA is an emerging field of study and their involvement in inducing or inhibiting inflammatory responses via regulating NLRP3 inflammasome activity is not completely understood. Not only NLRP3 inflammasome inhibitors, but lncRNA regulators also prove as an attractive strategy to control NLRP3 inflammasome activity. Herbal derivatives or phytochemicals have been reported to be effective in regulating the activity of several lncRNAs in different types of cancers (186). However, lncRNA regulating phytochemicals directly affecting NLRP3 inflammasome activity has not yet been reported. Phytochemicals are components obtained from a variety of plants, vegetables, and fruits. They are non-nutritive, anti-oxidant, anti-inflammatory chemical constituents with reduced side effects. They are known to execute their function by altering several signaling pathways and by targeting specific molecular points (186).

Due to the potential role played by several lncRNAs in regulating the activity of NLRP3 inflammasome, targeting lncRNAs is a great approach to control NLRP3 inflammasome involved inflammatory conditions. Several phytochemicals are known to regulate the function of lncRNAs in cancer. For instance, camptothecin (CPT) (Camptotheca acuminate) (187, 188), curcumin (Curcuma longa) (189), 3,3′-diindolylmethane (DIM) (broccoli, cabbage and kale) (190–193), epigallocatechin-3-gallate (EGCG) (Almond and Green tea) (35, 194, 195), genistein (Soy products) (196–198), quercetin (Red grapes, broccoli and berries) (199–203) and resveratrol (Blueberries, mulberries, raspberries and grape skin) (204, 205). Although their direct contribution in regulating NLRP3 inflammasome linked lncRNAs are not reported, many of these compounds are known to regulate lncRNAs involved in the NLRP3 based signaling pathways. For instance, curcumin inhibits the expression of lncRNA GAS5 by intervening in the NF-kB, STAT-3 and Akt signaling pathway. Curcumin is also known to upregulate the expression of lncRNA H19, which inhibits the activation of p53. NF-kB and p53 are well-known factors in the activation of the NLRP3 inflammasome mediated inflammatory pathway. The phytochemical EGCG potentially reduces the expression of several non-coding RNAs involved in the NF-kB signaling pathway. Similarly, resveratrol is reported to inhibit the expression of lncRNA MALAT-1.

The first report on NLRP3 inflammasome controlling lncRNA regulating compound was reported recently by Han et al. (63). In their study, the authors demonstrated the potential of sinapic acid (SA) in controlling the pyroptotic death of BMDMs in the inflammatory condition, diabetic atherosclerosis (DA) (63). Sinapic acid (4-hydroxy-3,5-two methoxy cinnamic acid) is abundantly available in Sinalbin and Yellow mustard seeds. SA is considered a potential Chinese medicine due to its anti-inflammatory, anti-oxidative and analgesic properties (63). The administration of 50 mg/kg sinapic acid in DA induced rat models reduced the levels of IL-1β, endothelin (ET-1), caspase-1, ASC and NLRP3 in the serum. The dose-dependent reduction of blood glucose, inflammation, and associated pyroptosis in response to SA was reported in DA rat models (62). In the study conducted by Han et al. it was shown that sinapic acid reduces the inflammatory condition in diabetic atherosclerosis (DA) by suppressing the activity of lncRNA MALAT-1 (63). In Section LncRNA MALAT-1 described above, we explained the role of lncRNA MALAT-1 in promoting the inflammatory responses in macrophages by enhancing the expression of NLRP3 inflammasome. The upregulated expression of lncRNA MALAT-1 observed in macrophages derived from DA rats was reduced significantly after the administration of 1 μM SA. LncRNA MALAT-1 promoted the expression of the NLRP3 inflammasome by inhibiting the activity of miR-23C (Section LncRNA MALAT-1). The introduction of 1 μM SA released miR-23C from the inhibitory action of MALAT-1. Thus, the SA promoted levels of miR-23C exhibited its inhibitory action on NLRP3 inflammasome thereby, reducing the incidence of pyroptosis (63). The response of MALAT-1 levels to different doses of SA was coinciding with the SA induced reduction in cell pyroptosis. Hence, it was concluded that in DA induced BMDMs, SA altered the levels of oxidative stress marker, lactate dehydrogenase (LDH) and reduced pyroptosis by alleviating the activity of lncRNA MALAT-1 in a dose-dependent pattern (63).

Such abundant knowledge in regulating the action of lncRNAs by phyto compounds can be applied to develop novel strategies to target NLRP3 inflammasome activity at various levels to control a range of inflammatory conditions. However, due to certain limitations of phytochemicals such as restricted efficacy and reduced bioavailability, their modification with nanoparticles, liposomes or certain adjuvants is necessary to improve their application in targeting lncRNAs.

## Future Prospects of LncRNA Studies

Despite the complexities involved in understanding the consolidated mechanism underlying the activation, NLRP3 inflammasome is the most comprehensively probed inflammasome in the recent past. Numerous lncRNAs critically participate in the controlled function of NLRP3 inflammasome in a variety of disease progression. The dynamic and precise regulation of NLRP3 inflammasome associated gene expression during inflammatory responses makes lncRNAs suitable candidates to target. The flexibility offered by lncRNAs in their expression, shapes, sizes, locations and functional diversity benefit their interaction with the target molecules. With rising genomic research advancement, the identification of novel lncRNAs having significant function enhances our understanding of immune defense mechanisms at the molecular level. The effect of lncRNAs on NLRP3 inflammasome acts as an additional layer of gene expression regulation in transcriptional, post-transcriptional, translational and post-translation level. Even with the available technologies and methodologies, the complete regulatory function of lncRNAs on NLRP3 inflammasome has not been elucidated. Future investigations are required to have a revamped knowledge of lncRNA biology, molecular action, and targets to aid in the identification of unique and innovative therapeutic targets in restraining the activity of NLRP3 inflammasome. The possibility of controlling lncRNA expression and function by phytochemicals provide a novel insight on altering their effect in immune disorders. The rapid pace of lncRNA research worldwide ensures the unraveling of novel lncRNAs and their interacting targets in the field of molecular immunology in the near future.

## Author Contributions

K-FH is the guarantor of the article and conceived and designed the study. MM and K-FH wrote and finished the manuscript. All authors contributed to the article and approved the submitted version.

## Conflict of Interest

The authors declare that the research was conducted in the absence of any commercial or financial relationships that could be construed as a potential conflict of interest.

## References

[1] Kelley N Jeltema D Duan Y He Y. The NLRP3 inflammasome: an overview of mechanisms of activation and regulation. Int J Mol Sci. (2019) 20:3328. 10.3390/ijms20133328 31284572 PMC6651423

[2] Swanson KV Deng M Ting JPY. The NLRP3 inflammasome: molecular activation and regulation to therapeutics. Nat Rev Immunol. (2019) 33:477–89. 10.1038/s41577-019-0165-0 31036962 PMC7807242

[3] Yang Y Wang H Kouadir M Song H Shi F. Recent advances in the mechanisms of NLRP3 inflammasome activation and its inhibitors. Cell Death Dis. (2019) 10:1–1. 10.1038/s41419-019-1413-8 30755589 PMC6372664

[4] Fink SL Cookson BT. Apoptosis, pyroptosis, and necrosis: mechanistic description of dead and dying eukaryotic cells. Infect Immun. (2005) 73:1907–16. 10.1128/IAI.73.4.1907-1916.2005 15784530 PMC1087413

[5] Menu P Vince JE. The NLRP3 inflammasome in health and disease: the good, the bad and the ugly. Clin Exp Immunol. (2011) 166:1–15. 10.1111/j.1365-2249.2011.04440.x 21762124 PMC3193914

[6] Guo H Callaway JB Ting JP. Inflammasomes: mechanism of action, role in disease, and therapeutics. Nat Med. (2015) 21:677–87. 10.1038/nm.3893 26121197 PMC4519035

[7] Tartey S Kanneganti TD. Differential role of the NLRP3 inflammasome in infection and tumorigenesis. Immunology. (2019) 156:329–38. 10.1111/imm.13046 30666624 PMC6418426

[8] Di Virgilio F. The therapeutic potential of modifying inflammasomes and NOD-like receptors. Pharmacol Rev. (2013) 65:872–905. 10.1124/pr.112.006171 23592611

[9] Yao RW Wang Y Chen LL Cellular functions of long noncoding RNAs. Nat Cell Biol. (2019) 21:542–51. 10.1038/s41556-019-0311-8 31048766

[10] Fernandes JC Acuña SM Aoki JI Floeter-Winter LM Muxel SM. Long non-coding RNAs in the regulation of gene expression: physiology and disease. Non Coding RNA. (2019) 5:17. 10.3390/ncrna5010017 30781588 PMC6468922

[11] Quinn JJ Chang HY. Unique features of long non-coding RNA biogenesis and function. Nat Rev Genet. (2016) 17:47. 10.1038/nrg.2015.10 26666209

[12] Wilusz JE Freier SM Spector DL. 3′ end processing of a long nuclear-retained noncoding RNA yields a tRNA-like cytoplasmic RNA. Cell. (2008) 135:919–32. 10.1016/j.cell.2008.10.012 19041754 PMC2722846

[13] Hangauer MJ Vaughn IW McManus MT. Pervasive transcription of the human genome produces thousands of previously unidentified long intergenic noncoding RNAs. PLoS Genet. (2013) 9:e1003569. 10.1371/journal.pgen.1003569 23818866 PMC3688513

[14] Niemczyk M Ito Y Huddleston J Git A Abu-Amero S Caldas C. Imprinted chromatin around DIRAS3 regulates alternative splicing of GNG12-AS1, a long noncoding RNA. Am J Hum Genet. (2013) 93:224–35. 10.1016/j.ajhg.2013.06.010 23871723 PMC3738830

[15] Ziegler C Kretz M. The more the merrier—complexity in long non-coding rna loci. Front Endocrinol. (2017) 8:90. 10.3389/fendo.2017.00090 28487673 PMC5403818

[16] Sunwoo H Dinger ME Wilusz JE Amaral PP Mattick JS Spector DL. MEN ε/β nuclear-retained non-coding RNAs are up-regulated upon muscle differentiation and are essential components of paraspeckles. Genome Res. (2009) 19:347–59. 10.1101/gr.087775.108 19106332 PMC2661813

[17] Salzman J Gawad C Wang PL Lacayo N Brown PO. Circular RNAs are the predominant transcript isoform from hundreds of human genes in diverse cell types. PLoS ONE. (2012) 7:e30733. 10.1371/journal.pone.0030733 22319583 PMC3270023

[18] Memczak S Jens M Elefsinioti A Torti F Krueger J Rybak A. Circular RNAs are a large class of animal RNAs with regulatory potency. Nature. (2013) 495:333–8. 10.1038/nature11928 23446348

[19] Zhang Y Zhang XO Chen T Xiang JF Yin QF Xing YH. Circular intronic long noncoding RNAs. Mol Cell. (2013) 51:792–806. 10.1016/j.molcel.2013.08.017 24035497

[20] Jiang S Cheng SJ Ren LC Wang Q Kang YJ Ding Y. An expanded landscape of human long noncoding RNA. Nucleic Acids Res. (2019) 47:7842–56. 10.1093/nar/gkz621 31350901 PMC6735957

[21] Rinn JL Chang HY. Genome regulation by long noncoding RNAs. Ann Rev Biochem. (2012) 81:145–66. 10.1146/annurev-biochem-051410-092902 22663078 PMC3858397

[22] Zhang X Rice K Wang Y Chen W Zhong Y Nakayama Y. Maternally expressed gene 3 (MEG3) noncoding ribonucleic acid: isoform structure, expression, and functions. Endocrinology. (2010) 151:939–47. 10.1210/en.2009-0657 20032057 PMC2840681

[23] Stojic L Niemczyk M Orjalo A Ito Y Ruijter AE Uribe-Lewis S. Transcriptional silencing of long noncoding RNA GNG12-AS1 uncouples its transcriptional and product-related functions. Nat Comm. (2016) 7:1–4. 10.1038/ncomms10406 26832224 PMC4740813

[24] Martianov I Ramadass A Barros AS Chow N Akoulitchev A. Repression of the human dihydrofolate reductase gene by a non-coding interfering transcript. Nature. (2007) 445:666–70. 10.1038/nature05519 17237763

[25] Beltran M Puig I Peña C García JM Álvarez AB Peña R. A natural antisense transcript regulates Zeb2/Sip1 gene expression during Snail1-induced epithelial–mesenchymal transition. Genes Dev. (2008) 22:756–69. 10.1101/gad.455708 18347095 PMC2275429

[26] Kondrashov AV Kiefmann M Ebnet K Khanam T Muddashetty RS Brosius J. Inhibitory effect of naked neural BC1 RNA or BC200 RNA on eukaryotic *in vitro* translation systems is reversed by poly (A)-binding protein (PABP). J Mol Biol. (2005) 353:88–103. 10.1016/j.jmb.2005.07.049 16154588

[27] Flynn RA Chang HY. Long noncoding RNAs in cell-fate programming and reprogramming. Cell Stem Cell. (2014) 14:752–61. 10.1016/j.stem.2014.05.014 24905165 PMC4120821

[28] Batista PJ Chang HY. Long noncoding RNAs: cellular address codes in development and disease. Cell. (2013) 152:1298–307. 10.1016/j.cell.2013.02.012 23498938 PMC3651923

[29] Wang KC Chang HY Molecular mechanisms of long noncoding RNAs. Mol cell. (2011) 43:904–14. 10.1016/j.molcel.2011.08.018 21925379 PMC3199020

[30] Ji P Diederichs S Wang W Böing S Metzger R Schneider PM. MALAT-1, a novel noncoding RNA, and thymosin beta4 predict metastasis and survival in early-stage non-small cell lung cancer. Oncogene. (2003) 22:8031–41. 10.1038/sj.onc.1206928 12970751

[31] Miyagawa R Tano K Mizuno R Nakamura Y Ijiri K Rakwal R. Identification of *cis*-and *trans*-acting factors involved in the localization of MALAT-1 noncoding RNA to nuclear speckles. RNA. (2012) 18:738–51. 10.1261/rna.028639.111 22355166 PMC3312561

[32] Tripathi V Ellis JD Shen Z Song DY Pan Q Watt AT. The nuclear-retained noncoding RNA MALAT1 regulates alternative splicing by modulating SR splicing factor phosphorylation. Mol Cell. (2010) 39:925–38. 10.1016/j.molcel.2010.08.011 20797886 PMC4158944

[33] Mathy NW Chen XM. Long non-coding RNAs (LncRNAs) and their transcriptional control of inflammatory responses. J Biol Chem. (2017) 292:12375–82. 10.1074/jbc.R116.760884 28615453 PMC5535013

[34] Ahmad I Valverde A Ahmad F Naqvi AR. Long noncoding RNA in myeloid and lymphoid cell differentiation, polarization and function. Cells. (2020) 9:269. 10.3390/cells9020269 31979061 PMC7072530

[35] Salviano-Silva A Lobo-Alves SC Almeida RCD Malheiros D Petzl-Erler ML. Besides pathology: long non-coding RNA in cell and tissue homeostasis. Non Coding RNA. (2018) 4:1–30. 10.3390/ncrna4010003 29657300 PMC5890390

[36] Li W Ren Y Si Y Wang F Yu J. Long non-coding RNAs in hematopoietic regulation. Cell Regener. (2018) 7:27–32. 10.1016/j.cr.2018.08.001 30671227 PMC6326246

[37] Atianand MK Caffrey DR Fitzgerald KA. Immunobiology of long noncoding RNAs. Ann Rev Immunol. (2017) 35:177–98. 10.1146/annurev-immunol-041015-055459 28125358 PMC6449690

[38] Geng H Tan XD. Functional diversity of long non-coding RNAs in immune regulation. Genes Dis. (2016) 3:72–81. 10.1016/j.gendis.2016.01.004 27617274 PMC5013731

[39] Yamazaki T Hirose T. The building process of the functional paraspeckle with long non-coding RNAs. Front Biosci. (2015) 7:1–41. 10.2741/e715 25553361

[40] Prasanth KV Prasanth SG Xuan Z Hearn S Freier SM Bennett CF. Regulating gene expression through RNA nuclear retention. Cell. (2005) 123:249–63. 10.1016/j.cell.2005.08.033 16239143

[41] Imamura K Imamachi N Akizuki G Kumakura M Kawaguchi A Nagata K. Long noncoding RNA NEAT1-dependent SFPQ relocation from promoter region to paraspeckle mediates IL8 expression upon immune stimuli. Mol Cell. (2014) 53:393–406. 10.1016/j.molcel.2014.01.009 24507715

[42] Wang Z Fan P Zhao Y Zhang S Lu J Xie W. NEAT1 modulates herpes simplex virus-1 replication by regulating viral gene transcription. Cell Mol Life Sci. (2017) 74:1117–31. 10.1007/s00018-016-2398-4 27783096 PMC5309293

[43] Wang Z Zhao Y Xu N Zhang S Wang S Mao Y. NEAT1 regulates neuroglial cell mediating Aβ clearance via the epigenetic regulation of endocytosis-related genes expression. Cell Mol Life Sci. (2019) 76:3005–18. 10.1007/s00018-019-03074-9 31006037 PMC6647258

[44] Cooper DR Carter G Li P Patel R Watson JE Patel NA. Long non-coding RNA NEAT1 associates with SRp40 to temporally regulate PPARγ2 splicing during adipogenesis in 3T3-L1 cells. Genes. (2014) 5:1050–63. 10.3390/genes5041050 25437750 PMC4276926

[45] Marchese A Raiborg C Santini F Keen JH Stenmark H Benovic JL The E3 ubiquitin ligase AIP4 mediates ubiquitination and sorting of the G protein-coupled receptor CXCR4. Dev Cell. (2003) 5:709–22. 10.1016/S1534-5807(03)00321-6 14602072

[46] Wang Z Li K Huang W. Long non-coding RNA NEAT1-centric gene regulation. Cell Mol Life Sci. (2020) 1-1. 10.1007/s00018-020-03503-0 32219465 PMC11104955

[47] Huang S Dong D Zhang Y Chen Z Geng J Zhao Y. NEAT1 regulates Th2 cell development by targeting STAT6 for degradation. Cell Cycle. (2019) 18:312–9. 10.1080/15384101.2018.1562285 30654703 PMC6380394

[48] Zhang P Cao L Zhou R Yang X Wu M. The LncRNA neat1 promotes activation of inflammasomes in macrophages. Nat Comm. (2019) 10:1–17. 10.1038/s41467-019-09482-6 30940803 PMC6445148

[49] Yu L Hao Y Xu C Zhu G Cai Y. LINC00969 promotes the degeneration of intervertebral disk by sponging miR-335-3p and regulating NLRP3 inflammasome activation. IUBMB Life. (2019) 71:611–8. 10.1002/iub.1989 30592131

[50] Paniri A Akhavan-Niaki H. Emerging role of IL-6 and NLRP3 inflammasome as potential therapeutic targets to combat COVID-19: role of lncRNAs in cytokine storm modulation. Life Sci. (2020) 257:118114. 10.1016/j.lfs.2020.118114 32693241 PMC7368418

[51] Xia LX Ke C Lu JM. NEAT1 contributes to neuropathic pain development through targeting miR-381/HMGB1 axis in CCI rat models. J Cell Physiol. (2018) 233:7103–11. 10.1002/jcp.26526 29633273 PMC13482416

[52] Dong P Xiong Y Yue J Hanley SJ Kobayashi N Todo Y. Long non-coding RNA NEAT1: a novel target for diagnosis and therapy in human tumors. Front Genet. (2018) 9:471. 10.3389/fgene.2018.00471 30374364 PMC6196292

[53] Zhang M Zheng Y Sun Y Li S Chen L Jin X. Knockdown of NEAT1 induces tolerogenic phenotype in dendritic cells by inhibiting activation of NLRP3 inflammasome. Theranostics. (2019) 9:3425–42. 10.7150/thno.33178 31281488 PMC6587165

[54] Arnold IC Zhang X Urban S Artola-Borán M Manz MG Ottemann KM. NLRP3 controls the development of gastrointestinal CD11b + dendritic cells in the steady state and during chronic bacterial infection. Cell Rep. (2017) 21:3860–72. 10.1016/j.celrep.2017.12.015 29281833

[55] Fernandez MV Miller E Krammer F Gopal R Greenbaum BD Bhardwaj N. Ion efflux and influenza infection trigger NLRP3 inflammasome signaling in human dendritic cells. J Leukoc Biol. (2016) 99:723–34. 10.1189/jlb.3A0614-313RRR 26574023 PMC4831479

[56] Yoshimoto R Mayeda A Yoshida M Nakagawa S. MALAT1 long non-coding RNA in cancer. Biochim Biophys Gene Regul Mech. (2016) 1859:192–9. 10.1016/j.bbagrm.2015.09.012 26434412

[57] Brown JA Bulkley D Wang J Valenstein ML Yario TA Steitz TA. Structural insights into the stabilization of MALAT1 noncoding RNA by a bipartite triple helix. Nat Struct Mol Biol. (2014) 21:633. 10.1038/nsmb.2844 24952594 PMC4096706

[58] Koshimizu TA Fujiwara Y Sakai N Shibata K Tsuchiya H. Oxytocin stimulates expression of a noncoding RNA tumor marker in a human neuroblastoma cell line. Life Sci. (2010) 86:455–60. 10.1016/j.lfs.2010.02.001 20149803

[59] Fan Y Shen B Tan M Mu X Qin Y Zhang F. TGF-β-induced upregulation of malat1 promotes bladder cancer metastasis by associating with suz12. Clin Cancer Res. (2014) 20:1531–41. 10.1158/1078-0432.CCR-13-1455 24449823

[60] Amodio N Raimondi L Juli G Stamato MA Caracciolo D Tagliaferri P. MALAT1: a druggable long non-coding RNA for targeted anti-cancer approaches. J Hematol Oncol. (2018) 11:63. 10.1186/s13045-018-0606-4 29739426 PMC5941496

[61] Yu SY Dong B Tang L Zhou SH. LncRNA MALAT1 sponges miR-133 to promote NLRP3 inflammasome expression in ischemia-reperfusion injured heart. Int J Cardiol. (2018) 254:50. 10.1016/j.ijcard.2017.10.071 29407129

[62] Li X Zeng L Cao C Lu C Lian W Han J. Long noncoding RNA MALAT1 regulates renal tubular epithelial pyroptosis by modulated MiR-23c targeting of ELAVL1 in diabetic nephropathy. Exp Cell Res. (2017) 350:327–35. 10.1016/j.yexcr.2016.12.006 27964927

[63] Han Y Qiu H Pei X Fan Y Tian H Geng J. Low-dose sinapic acid abates the pyroptosis of macrophages by downregulation of LncRNA-MALAT1 in rats with diabetic atherosclerosis. J Cardiovasc Pharmacol. (2018) 71:104–12. 10.1097/FJC.0000000000000550 29095793

[64] Bian Y Yang L Zhang B Li W Wang S Jiang S. LincRNA Cox-2 regulates lipopolysaccharide-induced inflammatory response of human peritoneal mesothelial cells via modulating miR-21/NF-κB Axis. Mediat Inflamm. (2019) 2019:1–11. 10.1155/2019/8626703 31885500 PMC6914883

[65] Ye Y Xu Y Lai Y He W Li Y Wang R. Long non-coding RNA cox-2 prevents immune evasion and metastasis of hepatocellular carcinoma by altering M1/M2 macrophage polarization. J Cell Biochem. (2017) 119:2951–63. 10.1002/jcb.26509 29131381

[66] Xue Z Zhang Z Liu H Li W Guo X Zhang Z. LincRNA-Cox2 regulates NLRP3 inflammasome and autophagy mediated neuroinflammation. Cell Death Differ. (2019) 26:130–45. 10.1038/s41418-018-0105-8 29666475 PMC6294802

[67] Guttman M Amit I Garber M French C Lin MF Feldser D Chromatin signature reveals over a thousand highly conserved large non-coding RNAs in mammals. Nature. (2009) 458:223–7. 10.1038/nature07672 19182780 PMC2754849

[68] Carpenter S Aiello D Atianand MK Ricci EP Gandhi P Hall LL. A long noncoding RNA mediates both activation and repression of immune response genes. Science. (2013) 341:789–92. 10.1126/science.1240925 23907535 PMC4376668

[69] Rao SS Huntley MH Durand NC Stamenova EK Bochkov ID Robinson JT. A 3D map of the human genome at kilobase resolution reveals principles of chromatin looping. Cell. (2014) 159:1665–80. 10.1016/j.cell.2014.11.021 25497547 PMC5635824

[70] Congrains A Kamide K Ohishi M Rakugi H. ANRIL: molecular mechanisms and implications in human health. Int J Mol Sci. (2013) 14:1278–92. 10.3390/ijms14011278 23306151 PMC3565320

[71] Kotake Y Nakagawa T Kitagawa K Suzuki S Liu N Kitagawa M. Long non-coding RNA ANRIL is required for the PRC2 recruitment to and silencing of p15 INK4B tumor suppressor gene.– Oncogene. (2011) 30:1956–62. 10.1038/onc.2010.568 21151178 PMC3230933

[72] Congrains A Kamide K Katsuya T Yasuda O Oguro R Yamamoto K. CVD-associated non-coding RNA, ANRIL, modulates expression of atherogenic pathways in VSMC. Biochem Biophys Res Comm. (2012) 419:612–6. 10.1016/j.bbrc.2012.02.050 22382030

[73] Kong Y Hsieh CH Alonso LC. ANRIL: a lncRNA at the CDKN2A/B locus with roles in cancer and metabolic disease. Front Endocrinol. (2018) 9:405. 10.3389/fendo.2018.00405 30087655 PMC6066557

[74] Kang YH Kim D Jin EJ. Down-regulation of phospholipase D stimulates death of lung cancer cells involving up-regulation of the long NcRNA ANRIL. Anticancer Res. (2015) 35:2795–803. 25964559

[75] Py BF Kim MS Vakifahmetoglu-Norberg H Yuan J. Deubiquitination of NLRP3 by BRCC3 critically regulates inflammasome activity. Mol Cell. (2013) 49:331–8. 10.1016/j.molcel.2012.11.009 23246432

[76] Hu W Yuan B Flygare J Lodish HF. LncRNA ANRIL promotes NLRP3 inflammasome activation in uric acid nephropathy through MiR-122-5p/BRCC3 axis. Biochimie. (2019) 157:102–10. 10.1016/j.biochi.2018.10.011 30347231

[77] Meseure D Drak Alsibai K Nicolas A Bieche I Morillon A. Long noncoding RNAs as new architects in cancer epigenetics, prognostic biomarkers, and potential therapeutic targets. BioMed Res Int. (2015) 2015:1–14. 10.1155/2015/320214 26448935 PMC4584070

[78] Jin X Jin H Shi Y Guo Y Zhang H. Long non-coding RNA KCNQ1OT1 promotes cataractogenesis via MiR-214 and activation of the caspase-1 pathway. Cell Physiol Biochem. (2017) 42:295–305. 10.1159/000477330 28535504

[79] Qiao Q Wang F Gu Y LncRNA Gm4419 promotes the development of cardiac diseases in type 2 diabetic patients with diabetic nephropathy. Int J Diabetes Dev Ctries. (2019) 39:369–73. 10.1007/s13410-018-0690-6

[80] Wen Y Yu Y Fu X. LncRNA Gm4419 Contributes to OGD/R injury of cerebral microglial cells via IκB phosphorylation and NF-?B activation. Biochem Biophys Res Commun. (2017) 487:923–9. 10.1016/j.bbrc.2017.05.005 28476620

[81] Yi H Peng R Zhang LY Sun Y Peng HM Liu HD. LincRNA-Gm4419 knockdown ameliorates NF-?B/NLRP3 inflammasome-mediated inflammation in diabetic nephropathy. Cell Death Dis. (2017) 8:1–14. 10.1038/cddis.2016.451 28151474 PMC5386454

[82] Lu KH Li W Liu XH Sun M Zhang ML Wu WQ. Long non-coding RNA MEG3 inhibits NSCLC cells proliferation and induces apoptosis by affecting p53 expression. BMC Cancer. (2013) 13:461. 10.1186/1471-2407-13-461 24098911 PMC3851462

[83] Hajjari M Khoshnevisan A Shin YK. Long non-coding RNAs in hematologic malignancies: road to translational research. Front Genet. (2013) 4:250. 10.3389/fgene.2013.00250 24312125 PMC3834238

[84] Zhang Y Liu X Bai X Lin Y Li Z Fu J. Melatonin prevents endothelial cell pyroptosis via regulation of long noncoding RNA MEG3/MiR-223/NLRP3 axis. Int J Lab Hematol. (2016) 38:42–9. 10.1111/jpi.12449 26362346

[85] Hu W Yuan B Flygare J Lodish HF. Long noncoding RNA-mediated anti-apoptotic activity in murine erythroid terminal differentiation. Genes Dev. (2011) 25:2573–8. 10.1101/gad.178780.111 22155924 PMC3248679

[86] Agliano F Fitzgerald KA Vella AT Rathinam VA Medvedev AE. Long Non-coding RNA LincRNA-EPS Inhibits Host Defense Against Listeria monocytogenes infection. Front Cell Infect Microbiol. (2020) 9:481. 10.3389/fcimb.2019.00481 32039056 PMC6987077

[87] Mehmood H Marwat AD Khan NA. Invasive Listeria monocytogenes gastroenteritis leading to stupor, bacteremia, fever, and diarrhea: a rare life-threatening condition. J Investig Med High Impact Case Rep. (2017) 5:2324709617707978. 10.1177/2324709617707978 28540315 PMC5431493

[88] Atianand MK Hu W Satpathy AT Shen Y Ricci EP Alvarez-Dominguez JR. A long noncoding RNA lincRNA-EPS acts as a transcriptional brake to restrain inflammation. Cell. (2016) 165:1672–85. 10.1016/j.cell.2016.05.075 27315481 PMC5289747

[89] Lamkanfi M Dixit VM. Inflammasomes and their roles in health and disease. Annu Rev Cell Dev Biol. (2012) 28:137–61. 10.1146/annurev-cellbio-101011-155745 22974247

[90] Chutkow WA Patwari P Yoshioka J Lee RT. Thioredoxin-interacting protein (Txnip) is a critical regulator of hepatic glucose production. J Biol Chem. (2008) 283:2397–406. 10.1074/jbc.M708169200 17998203

[91] Ferreira NE Omae S Pereira A Rodrigues MV Miyakawa AA Campos LC. Thioredoxin interacting protein genetic variation is associated with diabetes and hypertension in the Brazilian general population. Atherosclerosis. (2012) 221:131–6. 10.1016/j.atherosclerosis.2011.12.009 22236479

[92] Hansmeier NR Widdershooven PJ Khani S Kornfeld JW. Rapid generation of long noncoding RNA knockout mice using CRISPR/Cas9 technology. Non Coding RNA. (2019) 5:12. 10.3390/ncrna5010012 30678101 PMC6468733

[93] Brocker CN Kim D Melia T Karri K Velenosi TJ Takahashi S Long non-coding RNA Gm15441 attenuates hepatic inflammasome activation in response to metabolic stress. bioRxiv. (2019) 675785 10.1101/675785 PMC7673042 33203882

[94] Hong K Xu G Grayson TB Shalev A. Cytokines regulate β-cell thioredoxin-interacting protein (TXNIP) via distinct mechanisms and pathways. J Biol Chem. (2016) 291:8428–39. 10.1074/jbc.M115.698365 26858253 PMC4861417

[95] Anthony TG Wek RC. TXNIP switches tracks toward a terminal UPR. Cell Metab. (2012) 16:135–7. 10.1016/j.cmet.2012.07.012 22883225

[96] Oslowski CM Hara T O'Sullivan-Murphy B Kanekura K Lu S Hara M. Thioredoxin-interacting protein mediates ER stress-induced β cell death through initiation of the inflammasome. Cell Metab. (2012) 16:265–73. 10.1016/j.cmet.2012.07.005 22883234 PMC3418541

[97] Agrelo R Souabni A Novatchkova M Haslinger C Leeb M Komnenovic V. SATB1 defines the developmental context for gene silencing by Xist in lymphoma and embryonic cells. Dev Cell. (2009) 16:507–16. 10.1016/j.devcel.2009.03.006 19386260 PMC3997300

[98] Weakley SM Wang H Yao Q Chen C. Expression and function of a large non-coding RNA gene XIST in human cancer. World J Surg. (2011) 35:1751–6. 10.1007/s00268-010-0951-0 21212949 PMC3275083

[99] Chang S Chen B Wang X Wu K Sun Y. Long non-coding RNA XIST regulates PTEN expression by sponging miR-181a and promotes hepatocellular carcinoma progression. BMC Cancer. (2017) 17:248. 10.1186/s12885-017-3216-6 28388883 PMC5383949

[100] Yao Y Ma J Xue Y Wang P Li Z Liu J. Knockdown of long non-coding RNA XIST exerts tumor-suppressive functions in human glioblastoma stem cells by up-regulating miR-152. Cancer Lett. (2015) 359:75–86. 10.1016/j.canlet.2014.12.051 25578780

[101] Zhang XT Pan SX Wang AH Kong QY Jiang KT Yu ZB. Long non-coding RNA (lncRNA) X-Inactive Specific Transcript (XIST) plays a critical role in predicting clinical prognosis and progression of colorectal cancer. Med Sci Monit Int Med J Exp Clin Res. (2019) 25:6429. 10.12659/MSM.915329 31452526 PMC6724558

[102] Ma M Pei Y Wang X Feng J Zhang Y Gao MQ. LncRNA XIST mediates bovine mammary epithelial cell inflammatory response via NF-?B/NLRP3 inflammasome pathway. Cell Prolif. (2019) 52:1–12. 10.1111/cpr.12525 30362186 PMC6430464

[103] Ruland J. Return to homeostasis: downregulation of NF-?B responses. Nat Immunol. (2011) 12:709–14. 10.1038/ni.2055 21772279

[104] Lai JL Liu YH Liu C Qi MP Liu RN Zhu XF. Indirubin inhibits LPS-induced inflammation via TLR4 abrogation mediated by the NF-KB and MAPK signaling pathways. Inflammation. (2017) 40:1–2. 10.1007/s10753-016-0447-7 27718095

[105] Zhang W Li X Xu T Ma M Zhang Y Gao MQ. Inflammatory responses of stromal fibroblasts to inflammatory epithelial cells are involved in the pathogenesis of bovine mastitis. Exp Cell Res. (2016) 349:45–52. 10.1016/j.yexcr.2016.09.016 27680776

[106] Zhao X Lacasse P. Mammary tissue damage during bovine mastitis: causes and control. J Anim Sci. (2008) 86 (13 Suppl):57–65. 10.2527/jas.2007-0302 17785603

[107] Rackham O Shearwood AMJ Mercer TR Davies SM Mattick JS Filipovska A. Long noncoding RNAs are generated from the mitochondrial genome and regulated by nuclear-encoded proteins. RNA. (2011) 17:2085–93. 10.1261/rna.029405.111 22028365 PMC3222122

[108] Cabili MN Dunagin MC McClanahan PD Biaesch A Padovan-Merhar O Regev A. Localization and abundance analysis of human LncRNAs at single-cell and single-molecule resolution. Genome Biol. (2015) 16:1–16. 10.1186/s13059-015-0586-4 25630241 PMC4369099

[109] Lubelsky Y Ulitsky I. Sequences enriched in alu repeats drive nuclear localization of long RNAs in human cells. Nature. (2018) 555:107–11. 10.1038/nature25757 29466324 PMC6047738

[110] Zhang B Gunawardane L Niazi F Jahanbani F Chen X Valadkhan S. A novel RNA motif mediates the strict nuclear localization of a long noncoding RNA. Mol Cell Biol. (2014) 34:2318–29. 10.1128/MCB.01673-13 24732794 PMC4054287

[111] Kertesz M Wan Y Mazor E Rinn JL Nutter RC Chang HY. Genome-wide measurement of RNA secondary structure in yeast. Nature. (2010) 467:103–7. 10.1038/nature09322 20811459 PMC3847670

[112] Yang CA Huang ST Chiang BL. Sex-dependent differential activation of NLRP3 and AIM2 inflammasomes in SLE macrophages. Rheumatology.(2015) 54:324–31. 10.1093/rheumatology/keu318 25161312

[113] Strickland FM Hewagama A Lu Q Wu A Hinderer R Webb R. Environmental exposure, estrogen and two X chromosomes are required for disease development in an epigenetic model of lupus. J Autoimmunity. (2012) 38:J135–43. 10.1016/j.jaut.2011.11.001 22142890 PMC3312994

[114] Zang Y Zhou X Wang Q Li X Huang H. LncRNA FIRRE/NF-KB feedback loop contributes to OGD/R injury of cerebral microglial cells. Biochem Biophys Res Comm. (2018) 501:131–8. 10.1016/j.bbrc.2018.04.194 29715458

[115] Zhao X Zhang C Hua M Wang R Zhong C Yu J. NLRP3 inflammasome activation plays a carcinogenic role through effector cytokine IL-18 in lymphoma. Oncotarget. (2017) 8:108571–83. 10.18632/oncotarget.21010 29312552 PMC5752465

[116] Saxena A Carninci P. Long non-coding RNA modifies chromatin: epigenetic silencing by long non-coding RNAs. Bioessays. (2011) 33:830–9. 10.1002/bies.201100084 21915889 PMC3258546

[117] Tong Q Gong AY Zhang XT Lin C Ma S Chen J. LincRNA-Cox2 modulates TNF-α-induced transcription of Il12b gene in intestinal epithelial cells through regulation of Mi-2/NuRD-mediated epigenetic histone modifications. FASEB J. (2016) 30:1187–97. 10.1096/fj.15-279166 26578685 PMC4750408

[118] Fan HY Trotter KW Archer TK Kingston RE. Swapping function of two chromatin remodeling complexes. Mol Cell. (2005) 17:805–15. 10.1016/j.molcel.2005.02.024 15780937

[119] Wang H Wang L Erdjument-Bromage H Vidal M Tempst P Jones RS Zhang Y. Role of histone H2A ubiquitination in Polycomb silencing. Nature. (2004) 431:873–8. 10.1038/nature02985 15386022

[120] Cao R Zhang YI. SUZ12 is required for both the histone methyltransferase activity and the silencing function of the EED-EZH2 complex. Mol Cell. (2004) 15:57–67. 10.1016/j.molcel.2004.06.020 15225548

[121] Wang L Brown JL Cao R Zhang Y Kassis JA Jones RS. Hierarchical recruitment of polycomb group silencing complexes. Mol Cell. (2004) 14:637–46. 10.1016/j.molcel.2004.05.009 15175158

[122] Shi Y Lan F Matson C Mulligan P Whetstine JR Cole PA. Histone demethylation mediated by the nuclear amine oxidase homolog LSD1. Cell. (2004) 119:941–53. 10.1016/j.cell.2004.12.012 15620353

[123] Tachibana M Sugimoto K Fukushima T Shinkai Y. Set domain-containing protein, G9a, is a novel lysine-preferring mammalian histone methyltransferase with hyperactivity and specific selectivity to lysines 9 and 27 of histone H3. J Biol Chem. (2001) 276:25309–17. 10.1074/jbc.M101914200 11316813

[124] Sleutels F Zwart R Barlow DP. The non-coding Air RNA is required for silencing autosomal imprinted genes. Nature. (2002) 415:810–3. 10.1038/415810a 11845212

[125] Rinn JL Kertesz M Wang JK Squazzo SL Xu X Brugmann SA. Functional demarcation of active and silent chromatin domains in human HOX loci by noncoding RNAs. Cell. (2007) 129:1311–23. 10.1016/j.cell.2007.05.022 17604720 PMC2084369

[126] Zhao J Sun BK Erwin JA Song JJ Lee JT. Polycomb proteins targeted by a short repeat RNA to the mouse X chromosome. Science. (2008) 322:750–6. 10.1126/science.1163045 18974356 PMC2748911

[127] Shin JY Fitzpatrick GV Higgins MJ. Two distinct mechanisms of silencing by the KvDMR1 imprinting control region. EMBO J. (2008) 27:168–78. 10.1038/sj.emboj.7601960 18079696 PMC2206141

[128] Pandey RR Mondal T Mohammad F Enroth S Redrup L Komorowski J. Kcnq1ot1 antisense noncoding RNA mediates lineage-specific transcriptional silencing through chromatin-level regulation. Mol Cell. (2008) 32:232–46. 10.1016/j.molcel.2008.08.022 18951091

[129] Obaid M Udden SN Deb P Shihabeddin N Zaki MH Mandal SS. LncRNA HOTAIR regulates lipopolysaccharide-induced cytokine expression and inflammatory response in macrophages. Sci Rep. (2018) 8:1–18. 10.1038/s41598-018-33722-2 30353135 PMC6199307

[130] Terranova R Yokobayashi S Stadler MB Otte AP van Lohuizen M Orkin SH. Polycomb group proteins Ezh2 and Rnf2 direct genomic contraction and imprinted repression in early mouse embryos. Dev Cell. (2008) 15:668–79. 10.1016/j.devcel.2008.08.015 18848501

[131] Nagano T Mitchell JA Sanz LA Pauler FM Ferguson-Smith AC Feil R. The Air noncoding RNA epigenetically silences transcription by targeting G9a to chromatin. Science. (2008) 322:1717–20. 10.1126/science.1163802 18988810

[132] Frevert CW Felgenhauer J Wygrecka M Nastase MV Schaefer L. Danger-associated molecular patterns derived from the extracellular matrix provide temporal control of innate immunity. J Histochem Cytochem. (2018) 66:213–27. 10.1369/0022155417740880 29290139 PMC5958376

[133] Dos Santos G Rogel MR Baker MA Troken JR Urich D Morales-Nebreda L. Vimentin regulates activation of the NLRP3 inflammasome. Nat Comm. (2015) 6:1–13. 10.1038/ncomms7574 25762200 PMC4358756

[134] Amir-Zilberstein L Ainbinder E Toube L Yamaguchi Y Handa H Dikstein R. Differential regulation of NF- B by elongation factors is determined by core promoter type. Mol Cell Biol. (2007) 27:5246–59. 10.1128/MCB.00586-07 17502349 PMC1951948

[135] Law C Cheung P Histone variants and transcription regulation. in Epigenetics: Development and Disease. Dordrecht: Springer (2013) 319–41. 10.1007/978-94-007-4525-4 23150257

[136] Vyleta ML Wong J Magun BE. Suppression of ribosomal function triggers innate immune signaling through activation of the NLRP3 inflammasome. PLoS ONE. (2012) 7:e36044. 10.1371/journal.pone.0036044 22606244 PMC3351443

[137] Hirose T Virnicchi G Tanigawa A Naganuma T Li R Kimura H. NEAT1 long noncoding RNA regulates transcription via protein sequestration within subnuclear bodies. Mol Biol Cell. (2014) 25:169–83. 10.1091/mbc.e13-09-0558 24173718 PMC3873887

[138] Lin L Xu L Lv W Han L Xiang Y Fu L. An NLRP3 inflammasome-triggered cytokine storm contributes to streptococcal toxic shock-like syndrome (STSLS). PLoS Pathog. (2019) 15:e1007795. 10.1371/journal.ppat.1007795 31170267 PMC6553798

[139] Tezcan G Martynova EV Gilazieva ZE McIntyre A Rizvanov AA Khaiboullina SF. MicroRNA post-transcriptional regulation of the NLRP3 inflammasome in immunopathologies. Front Pharmacol. (2019) 10:1–22. 10.3389/fphar.2019.00451 31118894 PMC6504709

[140] Hadjicharalambous MR Roux BT Feghali-Bostwick CA Murray LA Clarke DL Lindsay MA. Long non-coding RNAs are central regulators of the IL-1β-induced inflammatory response in normal and idiopathic pulmonary lung fibroblasts. Front Immunol. (2018) 9:2906. 10.3389/fimmu.2018.02906 30619270 PMC6299252

[141] Yang M Sun Y Xiao C Ji K Zhang M He N. Integrated analysis of the altered LncRNAs and MRNAs expression in 293T cells after ionizing radiation exposure. Int J Mol Sci. (2019) 20:2968. 10.3390/ijms20122968 31216644 PMC6627384

[142] Chaudhary R Lal A. Long noncoding RNAs in the P53 network. Physiol Behav. (2017) 8:e1410. 10.1002/wrna.1410 27990773 PMC6314037

[143] Licandro G Ling Khor H Beretta O Lai J Derks H Laudisi F. The NLRP3 inflammasome affects DNA damage responses after oxidative and genotoxic stress in dendritic cells. Eur J Immunol. (2013) 43:2126–37. 10.1002/eji.201242918 23619996

[144] Yoon J Abdelmohsen K Srikantan S Yang X Martindale JL De S Errata LincRNA-P21 suppresses target MRNA translation repression of the long noncoding RNA-LET by histone deacetylase 3 contributes to hypoxia-mediated metastasis. Mol Cell. (2013) 50:303–4. 10.1016/j.molcel.2013.04.008

[145] Zhang X Wang W Zhu W Dong J Cheng Y Yin Z. Mechanisms and functions of long non-coding RNAs at multiple regulatory levels. Int J Mol Sci. (2019) 20:5573. 10.3390/ijms20225573 31717266 PMC6888083

[146] Yoon JH Abdelmohsen K Srikantan S Yang X Martindale JL De S. LincRNA-p21 suppresses target mRNA translation. Mol Cell. (2012) 47:648–55. 10.1016/j.molcel.2012.06.027 22841487 PMC3509343

[147] Knap P Tebaldi T Di Leva F Biagioli M Dalla Serra M Viero G. The unexpected tuners: are lncRNAs regulating host translation during infections? Toxins. (2017) 9:357. 10.3390/toxins9110357 29469820 PMC5705972

[148] He RZ Luo DX Mo YY. Emerging roles of lncRNAs in the post-transcriptional regulation in cancer. Genes Dis. (2019) 6:6–15. 10.1016/j.gendis.2019.01.003 30906827 PMC6411652

[149] Zhou Y Zhang X Klibanski A MEG3 noncoding RNA: a tumor suppressor. J Mol Endocrinol. (2012) 48:R45–53. 10.1530/JME-12-0008 22393162 PMC3738193

[150] Sawicka K Bushell M Spriggs KA Willis AE. Polypyrimidine-tract-binding protein: a multifunctional RNA-binding protein. Biochem Soc Transact. (2008) 36:641–7. 10.1042/BST0360641 18631133

[151] Fontana MF Baccarella A Pancholi N Pufall MA De'Broski RH Kim CC. JUNB is a key transcriptional modulator of macrophage activation. Physiol Behav. (2017) 194:177–86. 10.4049/jimmunol.1401595 25472994 PMC4431620

[152] Shao BZ Cao Q Liu C. Targeting NLRP3 inflammasome in the treatment of CNS diseases. Front Mol Neurosci. (2018) 11:320. 10.3389/fnmol.2018.00320 30233319 PMC6131647

[153] An N Gao Y Zhang H Wang L Tian C Yuan M. Regulatory mechanisms of the NLRP3 inflammasome, a novel immune-inflammatory marker in cardiovascular diseases. Front Immunol. (2019) 10:1592. 10.3389/fimmu.2019.01592 31354731 PMC6635885

[154] Gross O Thomas CJ Guarda G Tschopp J. The inflammasome: an integrated view. Immunol Rev. (2011) 243:136–51. 10.1111/j.1600-065X.2011.01046.x 21884173

[155] Abais JM Xia M Zhang Y Boini KM Li PL Redox regulation of NLRP3 inflammasomes: ROS as trigger or effector?. Antioxidants Redox Signal. (2015) 22:1111–29. 10.1089/ars.2014.5994 PMC4403231 25330206

[156] Bartlett R Stokes L Sluyter R. The P2X7 receptor channel: recent developments and the use of P2X7 antagonists in models of disease. Pharmacol Rev. (2014) 66:638–75. 10.1124/pr.113.008003 24928329

[157] Yin J Wang Y Hu H Li X Xue M Cheng W. P2X7 receptor inhibition attenuated sympathetic nerve sprouting after myocardial infarction via the NLRP3/IL-1β pathway. J Cell Mol Med. (2017) 21:2695–710. 10.1111/jcmm.13185 28470940 PMC5661108

[158] Ward JR West PW Ariaans MP Parker LC Francis SE Crossman DC. Temporal interleukin-1β secretion from primary human peripheral blood monocytes by P2X7-independent and P2X7-dependent mechanisms. J Biol Chem. (2010) 285:23147–58. 10.1074/jbc.M109.072793 20495003 PMC2906308

[159] Mezzaroma E Toldo S Farkas D Seropian IM Van Tassell BW Salloum FN. The inflammasome promotes adverse cardiac remodeling following acute myocardial infarction in the mouse. Proc Natl Acad Sci USA. (2011) 108:19725–30. 10.1073/pnas.1108586108 22106299 PMC3241791

[160] Petrilli V Papin S Dostert C Mayor A Martinon F Tschopp J. Activation of the NALP3 inflammasome is triggered by low intracellular potassium concentration. Cell Death Differ. (2007) 14:1583–9. 10.1038/sj.cdd.4402195 17599094

[161] Halle A Hornung V Petzold GC Stewart CR Monks BG Reinheckel T. The NALP3 inflammasome is involved in the innate immune response to amyloid-β. Nat Immunol. (2008) 9:857. 10.1038/ni.1636 18604209 PMC3101478

[162] Hornung V Bauernfeind F Halle A Samstad EO Kono H Rock KL. Silica crystals and aluminum salts activate the NALP3 inflammasome through phagosomal destabilization. Nat Immunol. (2008) 9:847. 10.1038/ni.1631 18604214 PMC2834784

[163] Yuan X Bhat OM Meng N Lohner H Li PL. Protective role of autophagy in Nlrp3 inflammasome activation and medial thickening of mouse coronary arteries. Am J Pathol. (2018) 188:2948–59. 10.1016/j.ajpath.2018.08.014 30273598 PMC6334256

[164] Liu P Huang G Wei T Gao J Huang C Sun M. Sirtuin 3-induced macrophage autophagy in regulating NLRP3 inflammasome activation. Biochim Biophys Acta Mol Basis Dis. (2018) 1864:764–77. 10.1016/j.bbadis.2017.12.027 29277324

[165] Lai M Yao H Shah SZ Wu W Wang D Zhao Y. The NLRP3-caspase 1 inflammasome negatively regulates autophagy via TLR4-TRIF in prion peptide-infected microglia. Front Aging Neurosci. (2018) 10:116. 10.3389/fnagi.2018.00116 29720937 PMC5915529

[166] Liu C Wang J Yang Y Liu X Zhu Y Zou J. Ginsenoside Rd ameliorates colitis by inducing p62-driven mitophagy-mediated NLRP3 inflammasome inactivation in mice. Biochem Pharmacol. (2018) 155:366–79. 10.1016/j.bcp.2018.07.010 30012462

[167] Guo W Sun Y Liu W Wu X Guo L Cai P. Small molecule-driven mitophagy-mediated NLRP3 inflammasome inhibition is responsible for the prevention of colitis-associated cancer. Autophagy. (2014) 10:972–85. 10.4161/auto.28374 24879148 PMC4091180

[168] Lin YC Huang DY Wang JS Lin YL Hsieh SL Huang KC. Syk is involved in NLRP3 inflammasome-mediated caspase-1 activation through adaptor ASC phosphorylation and enhanced oligomerization. J Leukoc Biol. (2015) 97:825–35. 10.1189/jlb.3HI0814-371RR 25605870

[169] Stutz A Kolbe CC Stahl R Horvath GL Franklin BS Van Ray O. NLRP3 inflammasome assembly is regulated by phosphorylation of the pyrin domain. J Exp Med. (2017) 214:1725–36. 10.1084/jem.20160933 28465465 PMC5460996

[170] Rodgers MA Bowman JW Fujita H Orazio N Shi M Liang Q. The linear ubiquitin assembly complex (LUBAC) is essential for NLRP3 inflammasome activation. J Exp Med. (2014) 211:1333–47. 10.1084/jem.20132486 24958845 PMC4076580

[171] Ishii N Ozaki K Sato H Mizuno H Saito S Takahashi A. Identification of a novel non-coding RNA, MIAT, that confers risk of myocardial infarction. J Human Genet. (2006) 51:1087–99. 10.1007/s10038-006-0070-9 17066261

[172] Mackay D Coupe AM Shield J Storr J Temple I Robinson D. Relaxation of imprinted expression of ZAC and HYMAI in a patient with transient neonatal diabetes mellitus. Human Genet. (2002) 110:139–44. 10.1007/s00439-001-0671-5 11935319

[173] Chiesa N De Crescenzo A Mishra K Perone L Carella M Palumbo O. The KCNQ1OT1 imprinting control region and non-coding RNA: new properties derived from the study of Beckwith–Wiedemann syndrome and Silver–Russell syndrome cases. Human Mol Genet. (2012) 21:10–25. 10.1093/hmg/ddr419 21920939 PMC3235007

[174] Lin N Chang KY Li Z Gates K Rana ZA Dang J. An evolutionarily conserved long noncoding RNA TUNA controls pluripotency and neural lineage commitment. Mol Cell. (2014) 53:1005–19. 10.1016/j.molcel.2014.01.021 24530304 PMC4010157

[175] Graham LD Pedersen SK Brown GS Ho T Kassir Z Moynihan AT. Colorectal neoplasia differentially expressed (CRNDE), a novel gene with elevated expression in colorectal adenomas and adenocarcinomas. Genes Cancer. (2011) 2:829–40. 10.1177/1947601911431081 22393467 PMC3278902

[176] Parasramka MA Maji S Matsuda A Yan IK Patel T. Long non-coding RNAs as novel targets for therapy in hepatocellular carcinoma. Pharmacol Ther. (2016) 161:67–78. 10.1016/j.pharmthera.2016.03.004 27013343 PMC4851900

[177] Kimura T Aikata H Takahashi S Takahashi I Nishibuchi I Doi Y Stereotactic body radiotherapy for patients with small hepatocellular carcinoma ineligible for resection or ablation therapies. Hepatol Res. (2015) 45:378–86. 10.1111/hepr.12359 24849379

[178] Takahashi H Carninci P. Widespread genome transcription: new possibilities for RNA therapies. Biochem Biophys Res Comm. (2014) 452:294–301. 10.1016/j.bbrc.2014.08.139 25193698

[179] Lebo KJ Niederer RO Zappulla DC. A second essential function of the Est1-binding arm of yeast telomerase RNA. RNA. (2015) 21:862–76. 10.1261/rna.049379.114 25737580 PMC4408794

[180] Yang G Lu X Yuan L. LncRNA: a link between RNA and cancer. Biochim Biophys Acta Gene Regul Mech. (2014) 1839:1097–109. 10.1016/j.bbagrm.2014.08.012 25159663

[181] Pavco PA Bouhana KS Gallegos AM Agrawal A Blanchard KS Grimm SL. Antitumor and antimetastatic activity of ribozymes targeting the messenger RNA of vascular endothelial growth factor receptors. Clin Cancer Res. (2000) 6:2094–103. 10815937

[182] Vitiello M Tuccoli A Poliseno L Long non-coding RNAs in cancer: implications for personalized therapy. Cell Oncol. (2015) 38:17–28. 10.1007/s13402-014-0180-x PMC13004270 25113790

[183] Tsai MC Spitale RC Chang HY. Long intergenic noncoding RNAs: new links in cancer progression. Cancer Res. (2011) 71:3–7. 10.1158/0008-5472.CAN-10-2483 21199792 PMC3057914

[184] Miller JC Holmes MC Wang J Guschin DY Lee YL Rupniewski I. An improved zinc-finger nuclease architecture for highly specific genome editing. Nat Biotechnol. (2007) 25:778–85. 10.1038/nbt1319 17603475

[185] Mizrahi A Czerniak A Levy T Amiur S Gallula J Matouk I. Development of targeted therapy for ovarian cancer mediated by a plasmid expressing diphtheria toxin under the control of H19 regulatory sequences. J Transl Med. (2009) 7:1–1. 10.1186/1479-5876-7-69 19656414 PMC2734756

[186] Saghafi T Taheri RA Parkkila S Zolfaghari Emameh R. Phytochemicals as modulators of long non-coding RNAs and inhibitors of cancer-related carbonic anhydrases. Int J Mol Sci. (2019) 20:2939. 10.3390/ijms20122939 31208095 PMC6627131

[187] Zeng CW Zhang XJ Lin KY Ye H Feng SY Zhang H. Camptothecin induces apoptosis in cancer cells via MicroRNA-125b-mediated mitochondrial pathways. Mol Pharmacol. (2012) 81:578–86. 10.1124/mol.111.076794 22252650

[188] Lin CJ Lin YL Luh F Yen Y Chen RM. Preclinical effects of CRLX101, an investigational camptothecin-containing nanoparticle drug conjugate, on treating glioblastoma multiforme via apoptosis and antiangiogenesis. Oncotarget. (2016) 7:42408–21. 10.18632/oncotarget.9878 27285755 PMC5173144

[189] Esatbeyoglu T Huebbe P Ernst IM Chin D Wagner AE Rimbach G. Curcumin-from molecule to biological function. Angew Chem Int Ed. (2012) 51:5308–32. 10.1002/anie.201107724 22566109

[190] Minich DM Bland JS. A review of the clinical efficacy and safety of cruciferous vegetable phytochemicals. Nutr Rev. (2007) 65:259–67. 10.1111/j.1753-4887.2007.tb00303.x 17605302

[191] White PT Subramanian C Motiwala HF Cohen MS. Natural withanolides in the treatment of chronic diseases. Adv Exp Med Biol. (2016) 928:329–73. 10.1007/978-3-319-41334-1_14 27671823 PMC7121644

[192] Jin H Park MH Kim SM. 3,3′-Diindolylmethane potentiates paclitaxel-induced antitumor effects on gastric cancer cells through the Akt/FOXM1 signaling cascade. Oncol Rep. (2015) 33:2031–6. 10.3892/or.2015.3758 25633416

[193] Cai H Chen J He B Li Q Li Y Gao Y. A FOXM1 Related long non-coding RNA contributes to gastric cancer cell migration. Mol Cell Biochem. (2015) 406:31–41. 10.1007/s11010-015-2421-3 25907137

[194] Petric RC Braicu C Raduly L Zanoaga O Dragos N Monroig P. Phytochemicals modulate carcinogenic signaling pathways in breast and hormone-related cancers. OncoTargets Ther. (2015) 8:2053–66. 10.2147/OTT.S83597 26273208 PMC4532173

[195] Li GX Chen YK Hou Z Xiao H Jin H Lu G. Pro-Oxidative activities and dose-response relationship of (-)-Epigallocatechin-3-Gallate in the inhibition of lung cancer cell growth: a comparative study *in vivo* and *in vitro*. Carcinogenesis. (2010) 31:902–10. 10.1093/carcin/bgq039 20159951 PMC2864413

[196] Biersack B. Interactions between anticancer active platinum complexes and non-coding RNAs/MicroRNAs. Non Coding RNA Res. (2017) 2:1–17. 10.1016/j.ncrna.2016.10.001 30159416 PMC6096430

[197] Chen J Lin C Yong W Ye Y Huang Z. Calycosin and genistein induce apoptosis by inactivation of HOTAIR/p-Akt signaling pathway in human breast cancer MCF-7 cells. Cell Physiol Biochem. (2015) 35:722–8. 10.1159/000369732 25613518

[198] Chiyomaru T Fukuhara S Saini S Majid S Deng G Shahryari V. Long non-coding RNA Hotair is targeted and regulated by MIR-141 in Human cancer cells. J Biol Chem. (2014) 289:12550–65. 10.1074/jbc.M113.488593 24616104 PMC4007447

[199] Russo M Spagnuolo C Tedesco I Bilotto S Russo GL. The flavonoid quercetin in disease prevention and therapy: facts and fancies. Biochem Pharmacol. (2012) 83:6–15. 10.1016/j.bcp.2011.08.010 21856292

[200] Sudan S Rupasinghe HV. Quercetin-3-O- glucoside induces human DNA topoisomerase II inhibition, cell cycle arrest and apoptosis in hepatocellular carcinoma cells. Anticancer Res. (2014) 34:1691–700. 24692698

[201] Zhao JL Zhao J Jiao HJ. Synergistic growth-suppressive effects of quercetin and cisplatin on HepG2 human hepatocellular carcinoma cells. Appl Biochem Biotechnol. (2014) 172:784–91. 10.1007/s12010-013-0561-z 24122665

[202] Pan F Zhu L Lv H Pei C. Quercetin promotes the apoptosis of fibroblast-like synoviocytes in rheumatoid arthritis by upregulating LncRNA MALAT1. Int J Mol Med. (2016) 38:1507–14. 10.3892/ijmm.2016.2755 28026003

[203] Pratheeshkumar P Son YO Divya SP Wang L Turcios L Roy RV. Quercetin inhibits Cr(VI)-induced malignant cell transformation by targeting MiR-21-PDCD4 signaling pathway. Oncotarget. (2017) 8:52118–31. 10.18632/oncotarget.10130 28881718 PMC5581017

[204] Ji Q Liu X Fu X Zhang L Sui H Zhou L. Resveratrol inhibits invasion and metastasis of colorectal cancer cells via MALAT1 mediated Wnt/β-catenin signal pathway. PLoS ONE. (2013) 8:e78700. 10.1371/journal.pone.0078700 24244343 PMC3823921

[205] Tomita S Abdalla MOA Fujiwara S Matsumori H Maehara K Ohkawa Y. A cluster of noncoding RNAs activates the ESR1 locus during breast cancer adaptation. Nat Commun. (2015) 6:1–15. 10.1038/ncomms7966 25923108 PMC4421845

