# Supplementary material for: The Long Non-coding RNAs: Paramount Regulators of the NLRP3 Inflammasome
Source: Front Immunol. 2020 Sep 25;11:569524. doi: 10.3389/fimmu.2020.569524 (PMC7546312; doi:10.3389/fimmu.2020.569524)
Supplement: Supplementary file 1 [file Table_1.DOCX]

Table No. 1. Disease associated with NLRP3 inflammasome

| **S. No.** | **Disease Condition** | **NLRP3 Inflammasome Response** | **Mechanism** | ***In vitro /* *In vivo* Studies** | **Ref** |
| --- | --- | --- | --- | --- | --- |
| 1 | Head and Neck squamous cell carcinoma (HNSCC) | NLRP3 inflammasome activation | - Activation of purinergic receptor P2X7 - Secretion of IL-1β - Activation of cancer stem cells (CSC) - Self-renewal and progression of HNSCC | *In vitro* study:   - A253 cells from human squamous carcinoma in submandibular glands - HSG cells from human submandibular ducts | (Bae et al. 2017) |
| 2 | *H. pylori* induced gastric neoplasms | NLRP3 inflammasome activation | - Engages cyclic D1 - Maturation of IL-1β - Stimulation of JNK signalling cascade - Activation of NF-kB - Expression of IL-6, IL-18 and TNF-α - Differentiation, proliferation, invasion and development of gastric cancer cells | *In vitro* study*:*   - Murine BMDMs - Neutrophils from BMCs - Human PBMCs   *In vivo* study*:*   - C57BL/6 | (Semper et al. 2014)(Semper et al. 2014)(Hai Ping et al. 2016)(Bagheri et al. 2017)(Lamb and Chen 2013) |
| 3 | Gastric inflammation by *H. pylori* | NLRP3 inflammasome activation | - Down regulation of miR-22 - Secretion of IL-1β - Elevated expression of proliferation associated gene promoter CCND1 | *In vitro* study:   - AGS cells   *In vivo* study:   - C57BL/6 - Thymus null BALB/c nude mice | (Lamb and Chen 2013) |
| 4 | Inflammatory Bowel Disease induced Colorectal cancer | NLRP3 inflammasome inhibition | - Disrupt intestinal homeostasis - Absence of enterocyte differentiation - Interrupts the epithelial integrity in intestine - Increased permeability of epithelial barrier - Absence of IL-18 | - | (Franchi et al. 2006)(Franchi et al. 2006) |
| 5 | Liver colorectal cancer | NLRP3 inflammasome inhibition | - Inability of hepatic NK cell maturation - Inability to express the death ligand FasL - Absence of natural killer cells tumerocidal activity - Impaired IL-18 signalling pathway | *In vivo* study:   - C57BL/6 | (Dupaul-Chicoine et al. 2015) |
| 6 | Fulminant hepatic failures | NLRP3 inflammasome activation | - Upregulation of NLRP3, ASC, caspase-1 and TXNIP - Interaction of TXNIP with NLRP3 inflammasome links oxidative stress to NLRP3 inflammasome activation - Heme oxygenase-1 (HO-1) reversed the TXNIP-NLRP3 interaction - HO-1 interrupted NLRP3-ASC interaction - Increased levels of TNF-α and IL-1β | *In vivo* study:   - C57BL/6 | (Kim and Lee 2013) |
| 7 | Hepatic cell carcinoma (HCC) | NLRP3 inflammasome inhibition | - Low NLRP3 inflammasome proteins assist in HCC progression - Poor differentiation of cancer cell - Inability to respond to DAMP due to lack of NLRP3 inflammasome - Compromised cell survival | *In vitro* study:   - HCC cells - Non-cancerous liver cells | (Wei et al. 2014) |
| 8 | Oral squamous cell carcinoma (OSCC) | Enhanced expression of caspase-3 and caspase-9 | - Inhibitor of apoptosis protein cIAP2 inhibited activity of caspase-3 and 9 - Down regulation of cIAP2 increased caspase-3 and 9 expression - Enhanced apoptosis in OSCC | *In vitro* study:   - Human OSCC cell line derived from tongue tumor SAS | (Nagata et al. 2011)(Siegel et al. 2014)(Goldberg et al. 1997) |
| 9 | Lung adenocarcinoma | NLRP3 inflammasome activation | - High expression of IL-18 and IL-1β - Phosphorylation of intracellular kinases, Akt and ERK 1/2 - Phosphorylation and activation of transcription factor CREB - Enhanced expression of transcription factor snail to induce inflammation related cancer migration - Decreased expression of cellular adhesion molecule E-cadherin - Enhanced lung cancer cell migration and proliferation | *In vitro* study:   - A549 human alveolar epithelial adenocarcinoma cell line | (Y. Wang et al. 2016) |
| 10 | Pulmonary inflammation induced lung cancer | NLRP3 inflammasome activation and associated cytokine secretion | - Macrophages, neutrophils and eosinophils accumulation - Nickel oxide nanoparticle uptake and ROS induced NLRP3 inflammasome activation - Overexpression and activation of caspase-1 and release of IL-6, IL-1β and TNF-α | *In vitro* study:   - Murine macrophage cell line RAW 264.7   *In vivo* study:   - Sprague Dawley (SD) male adult rats | (Cao et al. 2016) |
| 11 | Lung inflammation and diseases (Mesothelioma) | Activation of NLRP3 inflammasome | - Activation of caspase-1 - Secretion of IL-1β - Induction of pyroptosis | - | (Sayan and Mossman 2016) |
| 12 | Breast neoplasm | Activation of NLRP3 inflammasome | - Secretion of IL-1β and IL-18 - Sphingolipid sphingosine- 1- phosphate signalling cascade initiation - Favourable microenvironment for the growth of breast cancer cells | *In vitro* study:   - Ductal carcinoma cells | (Balekouzou et al. 2016)(Kolb et al. 2014)(T. Wu et al. 2016) |
| 13 | Prostate cancer | NLRP3 inflammasome activation | - Destructive signals, stress, uric acid, urine crystals and infectious pathogens stimulate NLRP3 activation - Upregulated expression of NF-kB activates NLRP3 inflammasome - Stress in endoplasmic reticulum induces autophagy - Autophagy causes NLRP3 inflammasome activation - Hypoxia in prostate cells activated NLRP3 inflammasome | *In vitro* study:   - Prostate cell line BPH-1 and PC-3 | (C. S. Chen et al. 2013)(Veeranki 2013)(Panchanathan, Liu, and Choubey 2016) |
| 14 | Skin cancer | NLRP3 inflammasome activation | - Active Caspase-1 activity - Active IL-1β and IL-18 secretion - Activation of NF-kB - Presence of ROS | *In vitro* study:   - A375-Human melanoma cell line - B16F10-Mouse melanoma cell line   *In vivo* study:   - Wild-type female C57BL/6 | (Ahmad et al. 2013) |
| 15 | Cervical cancer | NLRP3 inflammasome activation | - Secretion of IL-1β and IL-18 | - | (Kriek et al. 2016)(Pontillo et al. 2009) |
| 16 | Glioblastoma | NLRP3 inflammasome activation | - Secretion of IL-1β - Activation of transcription factor STAT 3 - Enhanced neurotoxicity and angiogenesis | *In vitro* study:   - Glioblastoma cell lines U251 and U87 - Human umbilical vein endothelial cells (HUVEC-2) - Human fetal astrocytes | (Piñeros et al. 2016)(Tarassishin, Casper, and Lee 2014)(Moossavi et al. 2018) |
| 17 | Coronary atherosclerosis | NLRP3 inflammasome activation | - Endothelial cell injury - Activation of IL-1β, IL-18 and caspase-1 - cholesterol crystals/monosodium glutamate (MSG) uptake by macrophages via CD36 receptor - Lysosomal damage induce activation of NLRP3 inflammasome | *In vitro* study:   - Immortalised macrophage cell lines - Atherosclerotic BMDM models - Human PBMCs   *In vivo* study:   - Female *Ldlr-/-*C57BL/6 | (Bando et al. 2015)(Duewell et al. 2010)(Sheedy et al. 2013)(Shi et al. 2015)(He, Yang, and Peng 2012)(Okamura et al. 1998)(Mallat et al. 2001) |
| 18 | Cardiovascular diseases | NLRP3 inflammasome activation | - Secretion of mature IL-1β - Enhanced levels of thrombosis inducing substances and leukocyte adhesion molecules - IL-1β induce the release of adhesion molecules from vascular endothelial cells causing CVD | *In vitro* study:   - Human umbilical vein endothelial cells (HUVEC) - Human aortic endothelial cells (HAEC) - THP-1 human acute monocytic leukemia cells | (Libby 2017)(Shikama et al. 2015) |
| 19 | Coronary artery disease | NLRP3 inflammasome activation | - Secretion of mature IL-1β and IL-18 | *In vitro* study:   - PBMCs | (Satoh et al. 2014) |
| 20 | Atherosclerosis | NLRP3 inflammasome activation | - Mature caspase-1 induced the presence of atherosclerosis lesions - Enhanced cholesterol deposition due to absence of ATP binding cassette transporters A1 and G1 (Abca1/g1) - VECs induced injury | *In vivo* study:   - Caspase-1-null   (Casp1 -/-) C57BL/6 mice | (Gage et al. 2012)(Westerterp et al. 2018)(J. M. Wu et al. 2013)(Iyer et al. 2017) |
| 21 | Acute myocardial infarction (AMI) | NLRP3 inflammasome activation | - DAMPs secreted from ischemic injury and cell death - Release of pro-apoptotic proteins - Reperfusion induced mitochondrial injury and release of ROS - ROS causes vascular EC damage - Interaction of TXNIP with NLRP3 inflammasome | - | (Toldo and Abbate 2018)(Toldo et al. 2015)(Ibáñez et al. 2015)(Jong et al. 2014)(Liu et al. 2014)(Zhou and Chng 2013)(X. D. Wu et al. 2013)(Qian et al. 2010)(Gong et al. 2018)(Long et al. 2015)(Westermann et al. 2011)(C. Yao, Veleva, and Al 2018)(An et al. 2019) |
| 22 | Streptococcal toxic shock like syndrome (STSLS) | NLRP3 inflammasome activation | - Pore forming toxin from *S.suis,* suilysin causes membrane peforation - Cytosolic K+ efflux triggered NLRP3 inflammasome activation - Cytokine storm through activated caspase-1 | *In vitro* study:   - THP-1-nlrp3-/-cell line   *In vivo* study:   - 5 week-old Balb/c mice - nlrp3-/- C57BL/6 mice | (L. Lin et al. 2019) |
| 23 | Parkinson’s disease (PD) | NLRP3 inflammasome activation | - cytokine IL-1β/IL-18 triggered pyroptosis causes the death of dopamine neurons in substantia nigra - Downregulated expression of DRD1 in microglial cells - Dopamine neurons uses DRD1-cAMP pathway to inhibit the activation of NLRP3 inflammasome | *In vitro* study:   - BMDMs - Microglial cells   *In vivo* study:   - Nlrp3-/-, Drd1 -/- and Drd2 -/- mice | (S. Wang et al. 2019)(Guo, Callaway, and Ting 2015)(Yan et al. 2015) |
| 24 | Inflammatory kidney diseases | NLRP3 inflammasome activation | - Release of debris from cell apoptosis and necrosis activated NLRP3 inflammasome - Such cell debris included histones, high mobility group B1 proteins and heat shock proteins - Inflammasome stimulation occurred via TLR2 and TLR4 in a MyD88 dependent manner - Activation of NF-kB and associated secretion of pro-inflammatory cytokines | *In vitro* study:   - Mouse renal endothelial cells   *In vivo* study:   - C57BL/6J mice, 6–12 weeks old | (Allam et al. 2012)(Allam et al. 2013) |
| 25 | IgA nephropathy (IgAN) | NLRP3 inflammasome activation | - Increased ROS production - High levels of glomerular sclerosis, mesangial cell proliferation and lymphocyte infiltration | *In vitro* study:   - Murine macrophage cell line J774A.1   *In vivo* study:   - 8 weeks old female B cell-deficient mice (BCD) | (S. M. Yang et al. 2013)(Hua et al. 2013)(Tsai et al. 2017) |
| 26 | Inflammatory bowel disease (IBD) | NLRP3 inflammasome activation | - IL-1β increases the susceptibility to DSS-induced colitis - Aggravates the colonic damage by enhancing the inflammation - Ameliorates colitis damage in response to inflammation | *In vivo* study:   - DSS induced colitis mice model C57BL/6 | (Zhen and Zhang 2019) |
