# Supplementary material for: The Long Non-coding RNAs: Paramount Regulators of the NLRP3 Inflammasome
Source: Front Immunol. 2020 Sep 25;11:569524. doi: 10.3389/fimmu.2020.569524 (PMC7546312; doi:10.3389/fimmu.2020.569524)
Supplement: Supplementary file 2 [file Table_2.DOCX]

**Table 2: Summary of long non coding RNAs regulating NLRP3 inflammasome**

| **S.**  **No.** | **lncRNA** | **Abbreviation** | **Disease associated** | **Function** | **Mechanism** | **mRNA Sponging** | **NLRP3 inflammasome regulation** | **Ref** |
| --- | --- | --- | --- | --- | --- | --- | --- | --- |
| 1 | GM4419 | - | Diabetic retinopathy  Cerebral oxygen glucose deprivation/  reoxygenation damage (OGD/R injury) | Causes inflammation and fibrosis  Induces cell proliferation in mesangial cells  Induces the expression of pro-inflammatory cytokines in microglial cells | Interacts with P50 subunit of NF-kB  Causes IkB phosphorylation  Promotes nuclear translocation of p50/p65  Activates NF-kB  Causes inflammation through NF-kB/NLRP3 inflammasome axis | - | Up regulates NLRP3 inflammasome expression by simultaneous interaction with p50 unit and NLRP3 protein in mesangial cells | Yi et.al. 2017  Wen et. al. 2017 |
| 2 | MEG 3 | Maternally expressed gene 3 | Atherosclerosis | Causes pyroptosis in human aortic endothelial cells | Endogenously sponges and suppresses miR-223 function  Enhances NLRP3 expression  Increases pyroptosis of endothelial cells | miR-223 | Up regulates NLRP3 inflammasome expression  Enhances the mRNA and protein expression of NLRP3 and ASC in high fat diet mice model | Zhang et.al. 2016 |
| 3 | KCNQ10T1 | KCNQ overlapping transcript1/ KCNQ opposite strand anti-sense transcript 1 | Cataractogenesis | Promotes cataract formation  Promotes pyroptosis | Competitively binds miR-214  Activates caspase-1 pathway in human lens epithelial cells (HLECs) and SRA01/04 cells | miR-214 | Enhances NLRP3 inflammasome activity by promoting the mRNA and protein expression of caspase-1 in HLECs and SRA01/04 cells | Jin et. al. 2017 |
| 4 | ANRIL | Anti-sense non coding RNA in the INK locus | Uric acid nephropathy | Causes renal injury  Causes inflammation | Up regulates expression of deubiquitinating enzyme BRCC3  Sponges miR-122-5p  Increases the expression of pro-inflammatory cytokines | miR-122-5p | Up regulates NLRP3 inflammasome expression by enhancing the protein production of NLRP3, IL-18 and IL-1β in HK-2 cells | Hu et. al. 2018 |
| 5 | COX-2 | Cyclooxygenase-2 | Neuroinflammation | Induces expression of pro-inflammatory cytokines in BMDMs | Interacts with NF-kB subunit, p65  Promotes NF-kB nuclear translocation  Enhances TRIF cleavage and TRIF induced autophagy  Causes activation of NLRP3 inflammasome  Enhances caspase-1 activation | - | Up regulates NLRP3 inflammasome by promoting the expression of *nlrp3* and *Asc* mRNA in BV2 cells, BMDMs and microglial cells | Xue et. al. 2017 |
| 6 | MALAT 1 | Metastasis associated lung adenocarcinoma transcript 1 | Diabetic atherosclerosis | Activates pyroptosis of cells in diabetic atherosclerotic rats | Sponges miR-23C  Encourages the disengagement of miR-23C with ELAV1  Promotes activation of NLRP3 inflammasome | miR-23C | Up regulates NLRP3 inflammasome by enhancing the protein production of NLRP3, ASC, IL-1β, ELAVL1 and caspase-1 in BMDMs | Han et. al. 2018 |
| 7 | XIST | X-inactive specific transcript | Bovine mastitis | Enhances cell proliferation and viability  Reduces apoptosis in bovine mammary alveolar cell-T (MAC-T) epithelial cells | Suppresses NF-kB signalling cascade  Decreases the expression of pro-inflammatory cytokines | - | Suppresses NLRP3 inflammasome activity by reducing the protein production of NLRP3, pro-caspase 1 and ASC in MAC-T cells during inflammation | Ma et.al. 2018 |
| 8 | GM15441 |  | Hepatic inflammation and metabolic disorders | Assist in the activation of (PPARA)  Decreases the pro-inflammatory cytokine maturation in mouse liver | Suppresses the expression of *txnip*  Suppresses the activation of NLRP3 inflammasome  Reduces pro-caspase 1 and pro-IL-1β cleavage | - | Suppresses NLRP3 inflammasome activity by decreasing the protein expression of NLRP3, cleaved caspase-1and IL-1β in mice model | Brocker et. al. 2019 |
| 9 | NEAT-1 | Nuclear enriched abundant transcript 1 | Peritonitis  Pneumonia  Respiratory condition; COVID-19 | Regulates the expression of IL-6 | Interacts with pro-caspase-1  Stabilizes hetero tetramers of caspase-1  Enhances NLRP3 inflammasome function | miR-214 | Promotes NLRP3 inflammasome activity by stabilizing and enhancing the protease activity of caspase-1 in mice model | Wang et.al. 2020  Nie et. al. 2019 |
| 10 | EPS | Erythroid prosurvival | Inflammation | Regulate the expression of immune regulatory genes (IRGs) | Interacts with the regulatory regions present in the IRGs  Mediates positioning of nucleosome  Induces transcriptional repression  Interacts with the 3’ CANACA motif present in nucleo- riboprotein  Assist in the maintenance of repressed chromatin structure of IRGs | - | Suppress NLRP3 inflammasome activity by significantly reducing the production of ASC protein in BMDMs  Impaired production of IL-1β in BMDMs | Atianand et. al. 2016 |
