# Supplementary material for: The Long Non-coding RNAs: Paramount Regulators of the NLRP3 Inflammasome
Source: Front Immunol. 2020 Sep 25;11:569524. doi: 10.3389/fimmu.2020.569524 (PMC7546312; doi:10.3389/fimmu.2020.569524)
Supplement: Supplementary file 3 [file Table_3.DOCX]

**Table 3. Long non coding RNAs regulating NLRP3 inflammasome regulators**

| **S.**  **No.** | **lncRNA** | **Abbreviation** | **Inducer of lncRNA** | **NLRP3 inflammasome regulator** | **Effect of lncRNA on NLRP3 inflammasome regulator** | **Mechanism of action** | **Study model** | **Ref** |
| --- | --- | --- | --- | --- | --- | --- | --- | --- |
| 1 | HOTAIR | HOX antisense intergenic RNA | Ultra violet B rays | Double stranded RNA dependent protein kinase (PKR) | Enhances the expression of PKR | Phosphorylates IkBα, p65,AKT and p13K  Activates P13K/AKT pathway  Activate NF-kB pathway  Inhibits Bcl-2 protein  Increases IL-6 and TNF-α expression  Decreases cell viability | Human keratinocytes (HaCaT cells) | Liu and Zhang 2018 |
| 2 | Y00062 | - | - | NIMA related kinases 7 (NEK7) | Enhances the expression of NEK-7 | - | Mice  BMDMs | Xu et. al. 2015 |
| 3 | NEAT-1 | Nuclear enriched abundant transcript 1 | Hypoxia | Reactive oxygen species (ROS) | Enhances the production of ROS | HIF-2α dependent manner | C57BL/6 mice  Immortalized BMDMs | Zhang et. al. 2019  Bell et. al. 2007 |
| 4 | H19 | - | Mitochondrial membrane potential collapse | Intracellular ROS | Enhances the production of ROS | Promotes phosphorylation  Nuclear translocation of transcription factors  Enhances the transcription of target genes  Enhances caspase-1 cleavage  Promotes production of IL-1β and IL-18  Creates NLRP3/6 imabalance | C57BL/6 mice  Primary retinal microglial cells | Wan et. al. 2020 |
| 5 | KCNQ10T1 | KCNQ1 overlapping transcript 1 | L-OHP treatment | Autophagy | Induces autophagy | Up regulates the expression of Atg4B  Sponges miR-34a | Human colon cancer cell lines (HCT116 and SW480)  Male BALB/c nude mice | Li et.al. 2019 |
| 6 | HOTAIR M1 | HOX antisense intergenic RNA (located between HOXA1 and HOXA2) | Acute promyelocytic leukemia condition | Autophagy | Activation of autophagy | Regulates the expression of ATG and other adaptor proteins  Enhances formation of autophagosome  Increases protein expression of LC3B-II and GABARAP-II  Binds to AGO 2, a microRNA silencing complex component  Sponges miR-20a, miR-106b and miR-125b  Release E2F1, ULK1 and DRAM2  Regulates PML-RARA degradation | NB4, U937-PR9 cells and HEK-293T cells | Chen et.al.2017 |
| 7 | H19 | - | High glucose environment | Autophagy | Inhibits autophagy | Silences DIRAS3 epigenetically  Promotes phosphorylation of mTOR | Male Sprague-Dawley rats  Neonatal ventricular myocytes | Zhuo et. al.2017 |
| 8 | MEG-3 | Maternally expressed gene 3 | Bacillus Calmette -Guerin (BCG) infection | Autophagy | Inhibits autophagy | Regulates mTOR and P13K/AKT pathway  Decreases the levels of LC3A/B-II in the cell  Reduces p62 levels | THP-1 cells  (Human acute leukemia cell line) | Pawar et. al. 2016 |
